# An unusual thioredoxin system in the facultative parasite *Acanthamoeba castellanii*

**DOI:** 10.1007/s00018-021-03786-x

**Published:** 2021-02-18

**Authors:** David Leitsch, Alvie Loufouma Mbouaka, Martina Köhsler, Norbert Müller, Julia Walochnik

**Affiliations:** 1 Institute of Specific Prophylaxis and Tropical Medicine, Medical University of Vienna, Kinderspitalgasse 15, 1090 Vienna, Austria; 2 Institute of Parasitology, Vetsuisse Faculty, University of Bern, Länggassstrasse 122, 3012 Bern, Switzerland

**Keywords:** Thioredoxin reductase, Acanthamoeba castellanii, Redox system, Selenoprotein

## Abstract

The free-living amoeba *Acanthamoeba castellanii* occurs worldwide in soil and water and feeds on bacteria and other microorganisms. It is, however, also a facultative parasite and can cause serious infections in humans. The annotated genome of *A*. *castellanii* (strain Neff) suggests the presence of two different thioredoxin reductases (TrxR), of which one is of the small bacterial type and the other of the large vertebrate type. This combination is highly unusual. Similar to vertebrate TrxRases, the gene coding for the large TrxR in *A*. *castellanii* contains a UGA stop codon at the C-terminal active site, suggesting the presence of selenocysteine. We characterized the thioredoxin system in *A*. c*astellanii* in conjunction with glutathione reductase (GR), to obtain a more complete understanding of the redox system in *A*. *castellanii* and the roles of its components in the response to oxidative stress. Both TrxRases localize to the cytoplasm, whereas GR localizes to the cytoplasm and the large organelle fraction. We could only identify one thioredoxin (Trx-1) to be indeed reduced by one of the TrxRases, i.e., by the small TrxR. This thioredoxin, in turn, could reduce one of the two peroxiredoxins tested and also methionine sulfoxide reductase A (MsrA). Upon exposure to hydrogen peroxide and diamide, only the small TrxR was upregulated in expression at the mRNA and protein levels, but not the large TrxR. Our results show that the small TrxR is involved in the *A*. *castellanii*’s response to oxidative stress. The role of the large TrxR, however, remains elusive.

**Supplementary Information:**

The online version contains supplementary material available at 10.1007/s00018-021-03786-x.

## Introduction

*Acanthamoeba * spp. are ubiquitous soil amoebae which feed on bacteria and other microorganisms [1] but which are also facultative parasites in humans. Acanthamoebae mainly cause two distinct clinical manifestations: keratitis (*Acanthamoeba* keratitis, AK), occurring predominantly in contact lens wearers, and granulomatous encephalitis (granulomatous *Acanthamoeba encephalitis*, GAE), occurring almost exclusively in immunocompromised individuals. Although *Acanthamoeba* infections are relatively rare, they can be devastating, GAE being almost invariably fatal, and are difficult to treat. Infections are caused by several species or genotypes, respectively, of the genus *Acanthamoeba* [1]. Due to its long record as an object of study, *A*. *castellanii*, strain Neff, was chosen as the representative for the genus *Acanthamoeba* for a genome project [2] (see AmoebaDB).

The publication of the *A*. *castellanii* Neff genome has enabled directed searches for physiologically important enzymes and pathways including components of the thioredoxin and glutathione systems. In most organisms, the thioredoxin system and the glutathione system have numerous functions and jointly constitute a cellular redox network [3]. Both systems ultimately depend on NADPH as the principal reductant and, further, on disulfide reductases, i.e., thioredoxin reductase (TrxR) or glutathione reductase (GR), which harness FAD as a cofactor for the reduction of their substrates. The main substrates of TrxRases are thioredoxins which function as small electron carrier proteins [4] and can reduce a large number of targets, including essential enzymes such as ribonucleotide reductase, transcription factors, antioxidant enzymes such as peroxidases (Prx), and methionine sulfoxide reductases (Msr) [4]. The main substrate of GR is glutathione disulfide (GSSG) which is reduced to glutathione (GSH). Glutathione, in turn, can reduce a large number of targets in the cell. In fact, the role of the glutathione system is overlapping with the thioredoxin system and also comprises peroxidases and transcription factors. Due to their overlapping functions, the thioredoxin system and the glutathione system can partly substitute for each other but the loss of both pathways is highly deleterious to the cell [3]. The thioredoxin network in particular has been implied to have a role in many cellular processes related to cancer and other forms of disease [5]. It also constitutes a drug target in several pathogens, including *Plasmodium falciparum*, the causative agent of malaria [6], and *Entamoeba histolytica*, the causative agent of amebiasis [7].

Given our long-standing interest in the redox biology of protist parasites, we undertook a large-scale study on the redox network in *A*. *castellanii* Neff to identify its central factors, with a special emphasis on TrxR and its substrates.

## Results

### Identification redox factors in *A*. *castellanii*

The NCBI (https://www.ncbi.nlm.nih.gov) and AmoebaDB (https://amoebadb.org/amoeba) databases were searched to identify factors involved in the redox system of *A*. *castellanii*, strain Neff. The genome data predicted the presence of a varied NADPH-dependent redox system comprising two distinct thioredoxin reductases (Table 1), of which one is of the small bacterial type (henceforth referred to as Ac TrxR-S) and the other of the large vertebrate type (henceforth referred to as Ac TrxR-L). The closest homolog to Ac TrxR-S is thioredoxin-disulfide reductase in the spirochaete *Leptonema illini,* whereas the closest homolog to Ac TrxR-L is thioredoxin reductase-1 from the lancelet *Branchiostoma belcheri*. Amplification of both TrxR genes from cDNA was possible, confirming that both enzymes are expressed in *A*. *castellanii* Neff.

**Table 1:** Proteins under study

Data base entry	Acc. numbers (NCBI) (AmoebaDB)	Size (aa)	Additional information	Nearest structural homolog
Disulfide reductases				
Thioredoxin reductase 1, cytoplasmic, putative	XP_004353633 ACA_1153040	526	**Ac TrxR-L** in this study. Data base entry stops at UGA as a stop codon	Thioredoxin reductase-1 [*Branchiostoma belcheri*] (XP_019625027); 68% identities, 78% positives, 94% coverage
Thioredoxin-disulfide reductase	XP_004351681 ACA1_398900	321	**Ac TrxR-S** in this study	Thioredoxin-disulfide reductase [*Leptonema illini*] ( PKL33210); 67% identities, 83% positives, 99% coverage
Glutathione-disulfide reductase	XP_004338246 ACA1_336860	454	**Ac GR** in this study	glutathione-disulfide reductase [*Oceanibaculum indicum*] (WP_121217391); 64% identities, 78% positives, 98% coverage
Thioredoxins/thioredoxin-like proteins				
Thioredoxin-1, putative	XP_004335509 ACA_1246790	105	**Trx-1** in this study	Thioredoxin-2 [*Daphnia magna*] ( KZS05792); 62% identities, 78% positives, 100% coverage
Thioredoxin	XP_004351095 ACA1_109470	185	Presumptive misannotation	Not available
Thioredoxin, putative	XP_004340525 ACA1_045080	389	Presumptive misannotation. 103 aa within sequence can be amplified from cDNA, thioredoxin-like	Thioredoxin-like [*Galdireria sulfuraria*] ( XP_005704526); 34% identities, 52% positives, 51% coverage
Thioredoxin, putative	XP_004335504 ACA1_246540	718	Presumptive misannotation. 106 aa within sequence can be amplified from cDNA, thioredoxin-like	Hypothetical protein M758_N013200 [Ceratodon purpureus] (KAG0504854); 54% identities, 72% positives, 36% coverage
Thioredoxin-like 1, putative	XP_004349558 ACA1_322690	295	**Trx-2** in this study. PITH domain	Thioredoxin-like protein 1 [*Chrysochloris asiatica*] (XP_006837576); 50% identities, 68% positives, 97% coverage
Thioredoxin family protein 1, putative	XP_004341439 ACA1_265590	216	PITH domain	Thioredoxin-like protein 1 isoform X4 [*Homo sapiens*] 35% identities, 53% positives, 82% coverage
Thioredoxin domain containing protein	XP_004337209 ACA1_218400	119		Thioredoxin, mitochondrial-like [*Pomacea canaliculata*] (XP_025095496); 44% identities 67% positives, 87% coverage
Thioredoxin domain containing protein	XP_004351513 ACA1_149030	416	Presumptive misannotation	Not available
Peroxiredoxins				
Peroxiredoxin, putative	XP_004344639 ACA1_278470	199	**Prx-1** in this study	Peroxiredoxin [*Hyphomicrobium nitrativorans*] (WP_114356326); 60% identities, 72% positives, 93% coverage
Peroxiredoxin 2, putative	XP_004333640 ACA1_259510	199	**Prx-2** in this study	Peroxiredoxin-4-like [*Hydra vulgaris*] (XP_012555690); 70% identities, 82% positives, 100% coverage
Peroxiredoxin family protein	XP_004353768 ACA1_272630	250		Peroxiredoxin [Planctomycetes bacterium] (MBA2481489), 54% identities, 70% positives, 76% coverage
2-cys peroxiredoxin, putative	XP_004348542 ACA1_158790	217		Peroxiredoxin [*Aphanomyces invadans*] (XP_008878015), 70% identities, 84% positives, 88% coverage
Methioninesulfoxide reductases				
Peptidemethionine (S)-S-oxide reductase	XP_004342149 ACA1_113850	163	**MsrA** in this study	Peptide methionine sulfoxide reductase [*Lichtheimia corymbifera* JMRC:FSU:9682] (CDH51203); 64% identities, 76% positives, 100% coverage
methionineR-sulfoxide reductase	XP_004367953 ACA1_289310	139	**MsrB** in this study	Methionine sulfoxide reductase [*Catenaria anguillulae* PL171] (ORZ31325); 76% identities, 84% positives, 95% coverage

**Fig. 1:**
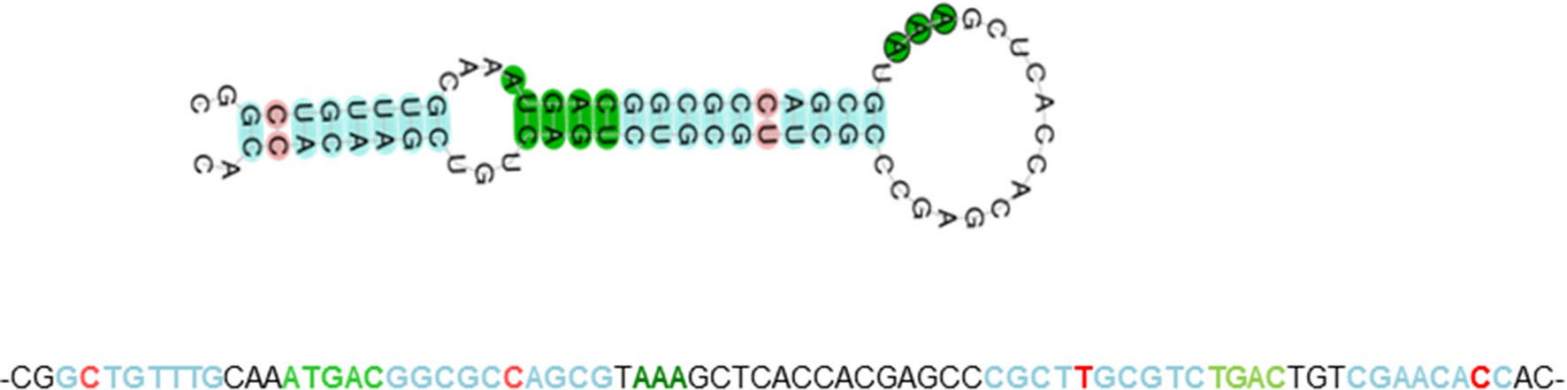
The predicted SECIS element in the 3′UTR of the TrxR-L gene in *A*. *castellanii* Neff. The predicted structure [13] is of type 1 and contains the typical helix–loop–helix structure with an AAA motif in the second loop [14]. Non-Watson–Crick pairings are also predicted to exist at critical positions such as the structurally relevant G-A tandems in the second helix. Below the predicted SECIS element, the corresponding DNA sequence in the 3′UTR is given. Nucleotides as highlighted in the image are given in the according colors

To the best of our knowledge, only one other genus of free-living but non-related amoebae, *Naegleria*, seems to have a similar combination. Other important representatives of the protist clade amoebozoa (which does include *Acanthamoeba* but not *Naegleria*), such as *Dictyostelium dendriticum* [8] and *E. histolytica* [9], only encode a TrxR of the small bacterial type. Large-type TrxRs also occur in insects [10] and some parasites, i.e., *P. falciparum* [11]. In contrast to these, but similar to the large TrxR in *Naegleria fowleri* [12], Ac TrxR-L is predicted to contain selenocysteine in its C-terminal active site through the presence of a pre-terminal UGA stop codon. This resembles TrxRases as found in higher eukaryotes [4]. The database entry for Ac TrxR-L (XP_004353633) stops at position 527 which is encoded by a UGA stop codon, and so the downstream DNA sequence (genomic scaffold NW_004457674) was searched for ensuing glycine and UAA stop codons as commonly found in this group of enzymes. Indeed, the UGA codon was found to be followed by a glycine codon (GGT) and a UAA stop codon and the 3′UTR of the gene was predicted by the selenoprotein prediction program Seblastian (https://seblastian.crg.es/) [13] to contain a selenocysteine identification sequence element (SECIS) of type 1 with typical features [14] at nt 282–355 downstream of the UAA stop codon (Fig. 1).

Furthermore, a selenocysteine tRNA is predicted to exist in *A*. *castellanii* according to RNA Central (https://rnacentral.org/rna/URS00006BD22B/1257118), as well as the putative selenocysteine-specific elongation factor SelB (XP_004346488), suggesting the existence of a functional insertion machinery for selenocysteine in *A*. *castellanii*.

The *A*. *castellanii* Neff genome further encodes a glutathione disulfide reductase (Ac GR) with extensive homology to proteobacterial GRs (Table 1). The respective gene was successfully amplified from cDNA by PCR. Several glutaredoxins and a glutathione peroxidase of the fungal type (Hyr1) were found in the genome but, given the focus of this study on the thioredoxin-related redox network in *A*. *castellanii*, not further considered.

Database searches also provided 19 proteins which were designated as thioredoxins or which comprise a thioredoxin domain, respectively (Table 1). Of these, 10 showed extensive homology to protein disulfide isomerases (PDI) or to disulfide bond formation protein A (DsbA) and were not further considered in this study (Supplementary Table 1). Another candidate which lacked a cysteine in its sequence was also omitted. Of the remaining candidates, four could be amplified from cDNA, two of which being standard size (ACA1_1246790, ACA1_218400) and the other two (ACA1_322690, ACA1_265590) being somewhat larger and harboring a “proteasome-interacting thioredoxin” (PITH) domain. The four remaining candidates could not be amplified from cDNA, and sequence alignments with BLAST strongly suggested that the genes had been incorrectly annotated because no full-length homologs were found. However, in three of these proteins (ACA1_045080, ACA1_246540, ACA1_149030) sections with extensive homologies to thioredoxins in other organisms could be identified. Strikingly, the complete sequence of one of the standard size thioredoxins (ACA_1246790) is contained in ACA1_246540 which further argues for the incorrect annotation of the latter. The two others contain thioredoxin-like polypeptides with homologies to thioredoxins from Atlantic herring and the fungus *Coccomyxa subellipsoidea* (Table 1). Both sequences were successfully amplified from *A*. *castellanii* Neff cDNA.

Four peroxiredoxins with homologies to either prokaryotic or eukaryotic peroxiredoxins were found (Table 1), all of which were expressed at the mRNA level. Three (ACA1_158790, ACA1_272630, ACA1_259510) are typical 2-Cys peroxiredoxins, and one is an atypical 2-Cys peroxiredoxin (ACA1_278470) of the bacterioferritin comigratory protein (BCP) subfamily type. Finally, two methionine sulfoxide reductases (Msr), one of the A-type and the B-type each (Table 1) were found in the genome. Also these genes could be amplified from cDNA.

### Recombinant expression of *A*. *castellanii* Neff redox factors

**Fig. 2:**
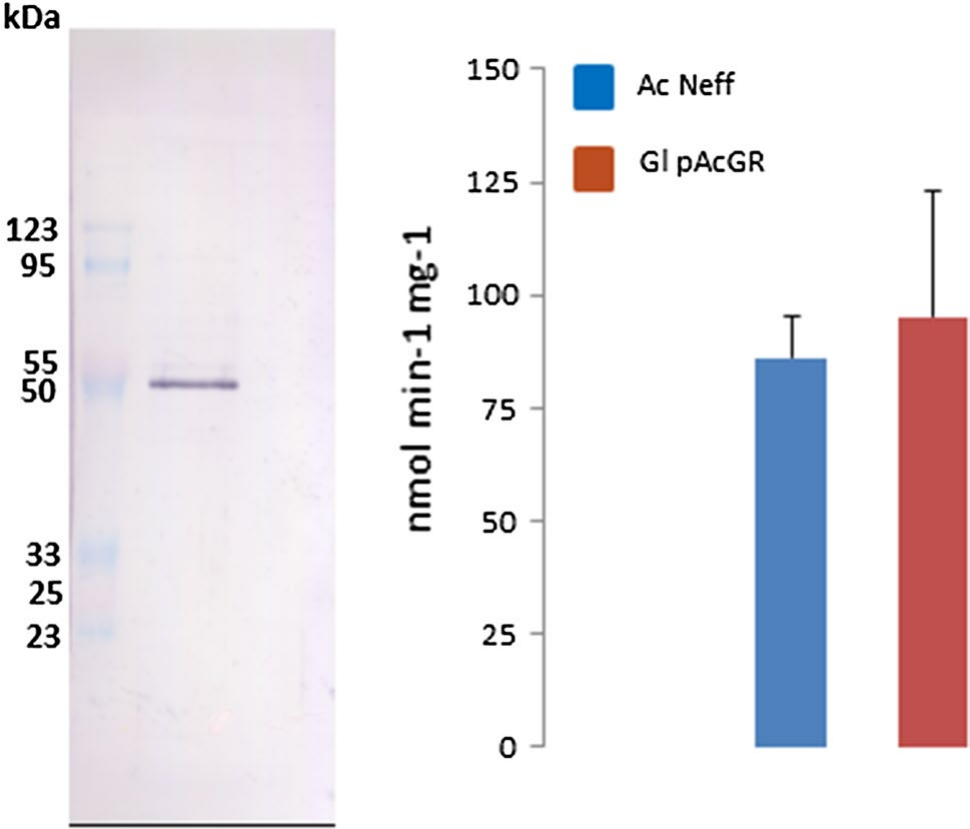
Expression of Ac GR in *G*. *lamblia*. Recombinant Ac GR was expressed in *G*. *lamblia* with the pPac-VInteg vector [31] and isolated in Ni-NTA agarose columns via its C-terminal 6 × histidine tag. The left panel shows a western blot with 10 µg of eluate and a polyclonal α-Ac GR serum (rabbit; dilution 1:2000). The predicted size of Ac GR based on its 454 aa and the 6 × histidine tag is approximately 49 kDa. The right panel shows Ac GR activity in transfected *G*. *lamblia* (mean and SD from 11 measurements) and in wild-type *A*. *castellanii* Neff (mean and SD from six measurements). Measurements were done at *λ* = 412 in 100 mM potassium phosphate buffer with 0.2 mM NADPH, 1 mM DTNB, 1 mM GSSG, and cell extract at the concentration of 50 µg protein ml^−1^. Values were read as reduction of DTNB by GSH, previously formed through reduction of GSSG by GSH

To confirm the predicted functions of the identified proteins in appropriate enzyme assays, their recombinant expression was attempted in *E*. *coli*. All genes that had been amplified from cDNA were cloned into expression vector pET-17b under addition of a C-terminal 6 × histidine tag for subsequent isolation in Ni-NTA-agarose columns. In case of most factors (Table 2), recombinant expression was conveniently achieved in *E*. *coli* BL21-AI™, a standard strain for recombinant protein expression with T7 RNA polymerase under the tight control of an *araB* promoter, released upon addition of L-arabinose to the growth medium (eluate fractions from recombinant proteins expressed in *E*. *coli* are shown in Supplementary Fig. 1). However, two peroxiredoxins (ACA1_278470 and ACA1_259510) could not be expressed under these conditions and required alternative *E*. *coli* strains specifically designed for the expression of redox sensitive proteins (Table 1) for successful expression. One thioredoxin candidate, ACA1_218400, could not be expressed at all and was synthesized by a biotechnology company (GenicBio, Shanghai, PR China). Further, recombinant Ac GR proved to be highly detrimental to *E*. *coli* and could not be introduced into any of the *E*. *coli* expression strains. Alternatively, recombinant Ac GR with a C-terminal 6 × histidine tag for subsequent purification was expressed from an appropriate plasmid in *Giardia lamblia* (Fig. 2). *G*. *lamblia* is a microaerophilic protist parasite which lacks glutathione [15] and, therefore, cannot be negatively affected by high copy numbers the enzyme as in *E*. *coli* and, arguably, in *A*. *castellanii* itself. Activity of recombinant Ac GR was confirmed by measuring its activity in cell extracts of transfected *G*. *lamblia* (Fig. 2). Interestingly, GR activity in transfected *G*. *lamblia* was very similar to that observed in extracts of *A*. *castellanii* Neff (Fig. 2).

**Table 2:** Recombinant proteins expressed in this study

Protein (Acc. Nr.)	Means of expression
Ac TrxR-L (ACA1_153040)	
Truncated, stops at UGA	*E*. *coli* BL21 AI™
C-terminal selenocysteine glycine (+ N-terminal 6 × His tag) + 3′ UTR with SECIS	*A*. *castellanii* Neff
C-terminal selenocysteine glycine (+ N-terminal 6 × His tag) + mutated 3′ UTR with inverted SECIS	*A*. *castellanii* Neff
Ac TrxR-S ( ACA1_398900)	*E*. *coli* BL21 AI™
Ac GR (ACA1_336860)	*G*. *lamblia* WB C6
Trx-1 (ACA1_246790)	*E*. *coli* BL21 AI™
Trx-2 ( ACA1_322690)	*E*. *coli* BL21 AI™
Thioredoxin candidate (ACA1_218400)	*E*. *coli* BL21 AI™
Thioredoxin candidate (ACA1_045080)	*E*. *coli* BL21 AI™
Thioredoxin candidate (ACA1_182230)	*E*. *coli* BL21 AI™
Prx-1 (ACA1_278470)	*E*. *coli* Origami™
Prx-2 (ACA1_259510)	*E*. *coli* SHuffle®
MsrA (ACA1_113850)	*E*. *coli* BL21 AI™
MsrB (ACA1_289310)	*E*. *coli* BL21 AI™

**Fig. 3:**
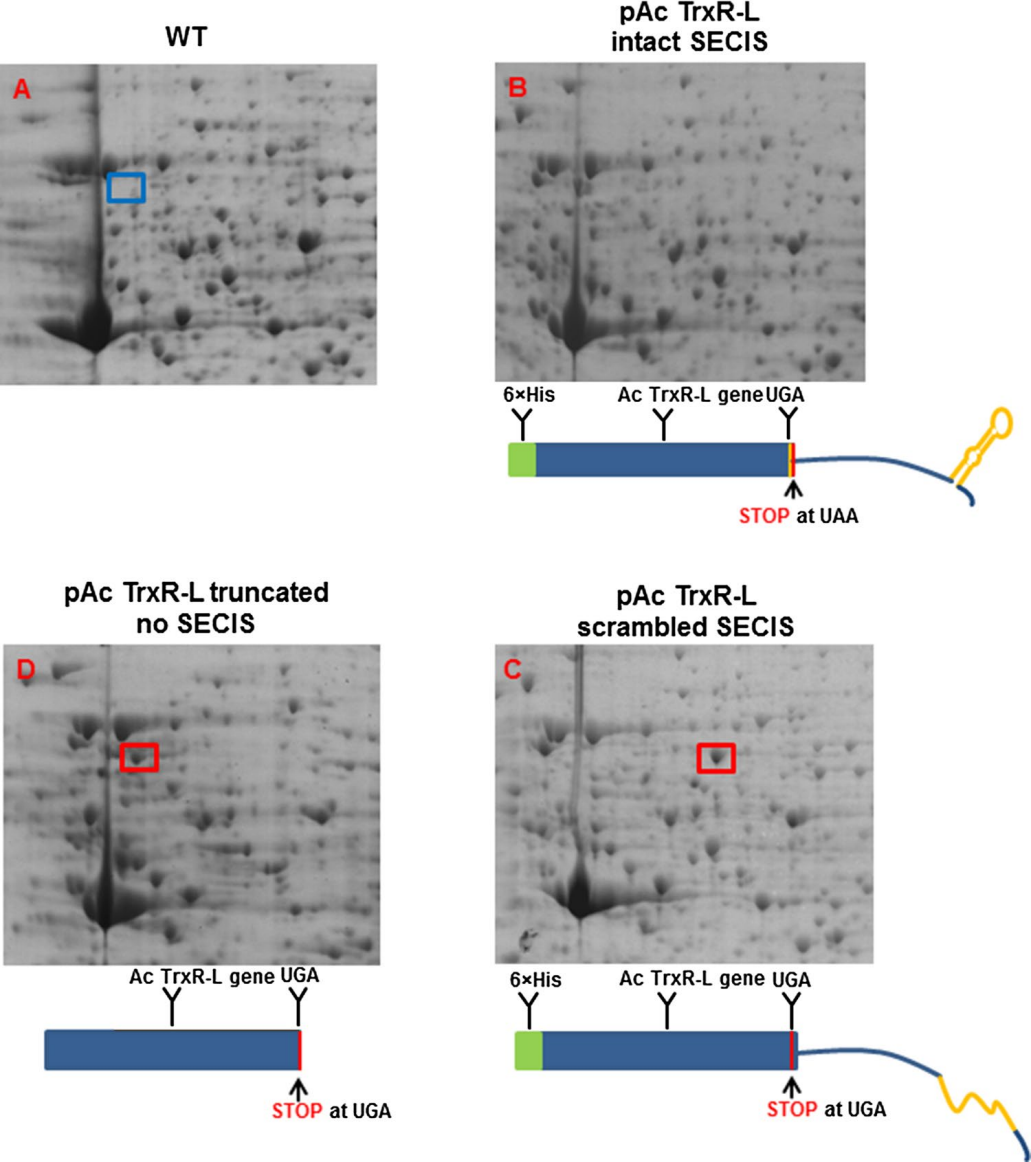
Recombinant expression of Ac TrxR-L in *A*. *castellanii* Neff. 2D-gels of *A*. *castellanii* Neff: cells were transfected with expression plasmids either carrying the Ac TrxR-L gene with an intact 3′UTR and an N-terminal 6 × His tag (**b**), an 3′ UTR with a scrambled SECIS (**c**) and an N-terminal 6 × His tag, or a truncated TrxR-L gene terminating at the UGA without an N-terminal 6 × His tag (**d**). **a**, 2D-gel of Neff control cells. Depictions of the gene constructs are given below the respective 2D-gels. Red rectangles indicate recombinant Ac TrxR-L without the terminal selenocysteine as expressed in transfected cell lines. Ac TrxR-L with 6 × His tag migrates towards a higher pI due to the positive charge of the additional histidines. The blue rectangle indicates the position of wild-type Ac TrxR-L in normal Neff cells

Expression of Ac TrxR-L was attempted in four different versions. In *E*. *coli*, Ac TrxR-L was expressed as a truncated polypeptide lacking the final two amino acids and, thus, terminating at the UGA stop codon. Further, it was attempted to express Ac TrxR-L in *E*. *coli* as a full-length peptide but with the UGA codon substituted for a cysteine codon. The second construct was designed because it had been reported that cysteine can partially substitute for selenocysteine in TrxR [16]. Unfortunately, however, this substitution rendered TrxR-L toxic for all *E*. *coli* expression strains at our disposal and no expression could be achieved. Due to this failure of expressing full-length Ac TrxR-L in *E*. *coli*, recombinant expression of two TrxR-L constructs was also performed via an appropriate plasmid [17] in *A*. *castellanii* Neff. The first construct comprised the gene together with 386 nt of the 3′UTR which contained the prospective SECIS element (Fig. 3b). The second construct comprised the gene and a mutated 3′UTR with an altered sequence and scrambled SECIS element (Fig. 3c). This construct was designed as a control for SECIS function. It was speculated that the original SECIS element would enable incorporation of selenocysteine in Ac TrxR-L; whereas, the mutated 3′UTR with the scrambled SECIS element would not. Both constructs also contained an N-terminal 6 × histidine tag for purification. The N-terminal position was chosen to avoid interference with the predicted C-terminal active site of Ac TrxR-L. Strikingly, only recombinant Ac TrxR-L which is encoded by the construct with the scrambled SECIS element could be expressed in *A*. *castellanii* to a noticeable extent as judged by 2D-gels from transfectant and control cells (Fig. 3c).

In fact, expression of the enzyme was very strong when the 3′-UTR had been scrambled, indicating that it was well tolerated by the cell. The identity of Ac TrxR-L was confirmed by mass spectrometric analysis of the isolated protein spot (Supplementary Fig. 2). However, the C-terminal peptide was not found among the peptides identified, arguably because the respective ion was not sufficiently stable to reach the detector of the mass spectrometer. Consequently, the C-terminal amino acid sequence could not be determined. Subsequently, Ac TrxR-L was isolated from transfected acanthamoebae via the N-terminal 6 × histidine tag. In contrast, no expression of recombinant Ac TrxR-L could be detected in *A*. *castellanii* Neff harboring the full-length construct with the intact SECIS (Fig. 3b). Also, the addition of 1 µM sodium selenite to the cultures did not result in any observable expression of Ac TrxR-L (not shown). Consequently, isolation of recombinant Ac TrxR-L from this transfectant cell line was unsuccessful. Taken together, these results suggest that an intact SECIS element is necessary for the insertion of the terminal selenocysteine and that high levels of Ac TrxR-L are tolerated by the cell if the terminal selenocysteine is missing but not if it is present.

It was also attempted to identify wild-type Ac TrxR-L in 2D-gels from *A*. *castellanii* Neff cell extracts to check if the selenocysteine residue would be included in the amino acid sequence. To that end, the position of TrxR-L was mapped by introduction of the same expression plasmid into *A*. *castellanii* Neff, but now encoding the TrxR-L gene terminating at the UGA and without the 6 × histidine tag. The resulting theoretical size and pI of the resulting polypeptide were practically identical to those of the wild-type enzyme and we hypothesized that it would migrate to the same position in 2D-gels. As expected, this truncated Ac TrxR-L was very strongly expressed from the plasmid construct in the transfectant cell line as indicated by 2DE (Fig. 3d), whereas in 2D-gels from wild-type cells no discernible spot could be found at the corresponding position (Fig. 3a). The protein spot of interest in the 2D-gel of the transfectant cell line and a gel patch at the corresponding position in the 2D-gel from wild-type *A*. *castellanii* Neff were both excised and submitted for mass spectrometric identification. Indeed, TrxR-L was identified in both samples, but again the final C-terminal peptide was missing from the number of identified peptides.

### Determination of enzymatic parameters

The isolated recombinant proteins were used for appropriate enzyme assays to confirm their proposed functions. Ac TrxR-L showed no observable activity with any of the thioredoxins tested, regardless of whether Ac TrxR-L had been expressed in *E*. *coli* or *A*. *castellanii* Neff, most likely due to the absence of the C-terminal selenocysteine. The enzyme also showed no activity with oxidized glutathione and almost no activity with DTNB. Thus, the function of Ac TrxR-L as a TrxR or as a disulfide reductase in general could not be confirmed.

In case of Ac TrxR-S, however, very strong activity was found with the thioredoxin ACA1_336860 and modest activity with thioredoxin ACA1_322690 (Table 3). All other thioredoxin candidates tested were not reduced by Ac TrxR-S. Based on these findings, ACA1_336860 will henceforth be referred to as Trx-1 and ACA1_322690 as Trx-2. Using Trx-1 as substrate, the sensitivity of TrxR-S to known inhibitors of TrxR activity, i.e., the drugs auranofin [18, 19] and aurothioglucose [20], was determined (Table 3). Both compounds inhibited Ac TrxR-S activity with an IC_50_ in the sub-micromolar range. Auranofin was similarly active against Ac TrxR-S as against TrxRases from other protist parasites [21–23] and aurothioglucose was similarly effective against Ac TrxR-S as against TrxR from rat liver [20]. Finally, Ac TrxR-S was found to have a very high affinity for NADPH (Table 3), as typically observed with TrxRases [3]. In contrast to GR activity which could be readily measured in cell extracts (Fig. 2), no TrxR activity in *A*. *castellanii* Neff cell extracts could be detected with any thioredoxin candidate tested.

**Table 3:** Enzymatic parameters of Ac TrxR-S and Ac GR

**Ac TrxR-S**						
Thioredoxin substrate	*v*_max_ (nmol min^−1^ mg^−1^)	*k*_cat_ (s^−1^)	Ratio** Trx:Trx-R** for 50% activity	*K*m NADPH	IC_50_ Auranofin	IC_50_ Aurothioglucose
Trx-1	2960	1.7	approx. 20:1	4 µM	216 nM	894 nM
Trx-2	390	0.22	approx. 7:1	ND	ND	ND

**Ac GR**	
*K*m GSSG	*K*m NADPH
93 µM	9 µM

*ND* not determined

**Fig. 4:**
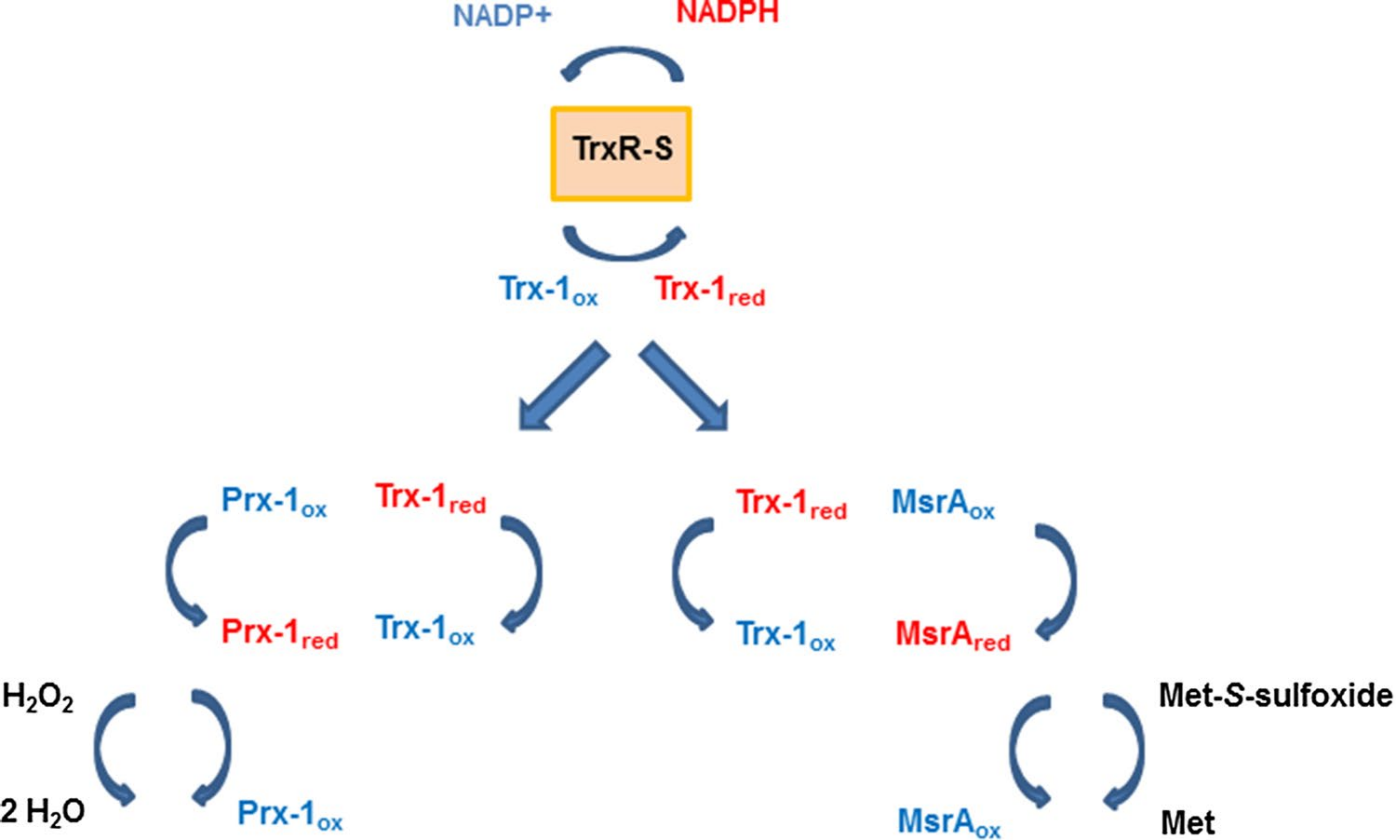
Components of the Trx pathway shown to be functional in this study. Ac TrxR-S harnesses NADPH to reduce Trx-1 which, in turn, reduces Prx-1 and Msr-A. Prx-1 reduces H_2_O_2_ to water; whereas, MsrA reduces methionine-*S*-sulfoxide to methionine in proteins affected by oxidation

The capacity of Trx-1 and Trx-2 to reduce the two expressed peroxiredoxins (henceforth designated as Prx-1 and Prx-2) and the two methionine sulfoxide reductases (according to their homologies MsrA and MsrB) was also evaluated. Trx-1 could reduce Prx-1, an untypical 2-Cys peroxiredoxin, and MsrA but not Prx-2, a typical 2-Cys peroxiredoxin, and MsrB (Table 4). Trx-2 did not reduce any of the factors assayed. To summarize, the existence of a Trx pathway in *A*. *castellanii*, at least consisting of TrxR-S, Trx-1, Prx-1, and MsrA, could be demonstrated (Fig. 4).

**Table 4:** Enzymatic parameters of Prx-1 and MsrA

Enzyme (substrate)	*K*m	*v*_max_	*k*_cat_	*K*_cat_/*K*m
**Prx-1** (H_2_O_2_)	26.4 µM	97 nmol min^−1^ mg^−1^	2.1 min^−1^	1326 M^−1^ s^−1^
**MsrA** (DL-methioninesulfoxide)	2.59 mM	500 nmol min^−1^ mg^−1^	9.3 min^−1^	59 M^−1^ s^−1^

Enzymes were reduced by Ac TrxR-S and Trx-1

Finally, also the Km of Ac GR with oxidized glutathione (GSSG) and NADPH were measured with the recombinant enzyme isolated from *G*. *lamblia* (Table 3). Ac GR showed typical characteristics as compared to other GRs [24]. Vmax was not measured because the eluate fraction also contained *G*. *lamblia* proteins (not shown) and the proportion of recombinant AC GR in the fraction could not be determined. Individual values of the kinetic analyses of all enzymes and inhibitors assayed can be found in Supplementary Table 2.

### Expression studies on thioredoxin reductases and glutathione disulfide reductase

**Fig. 5:**
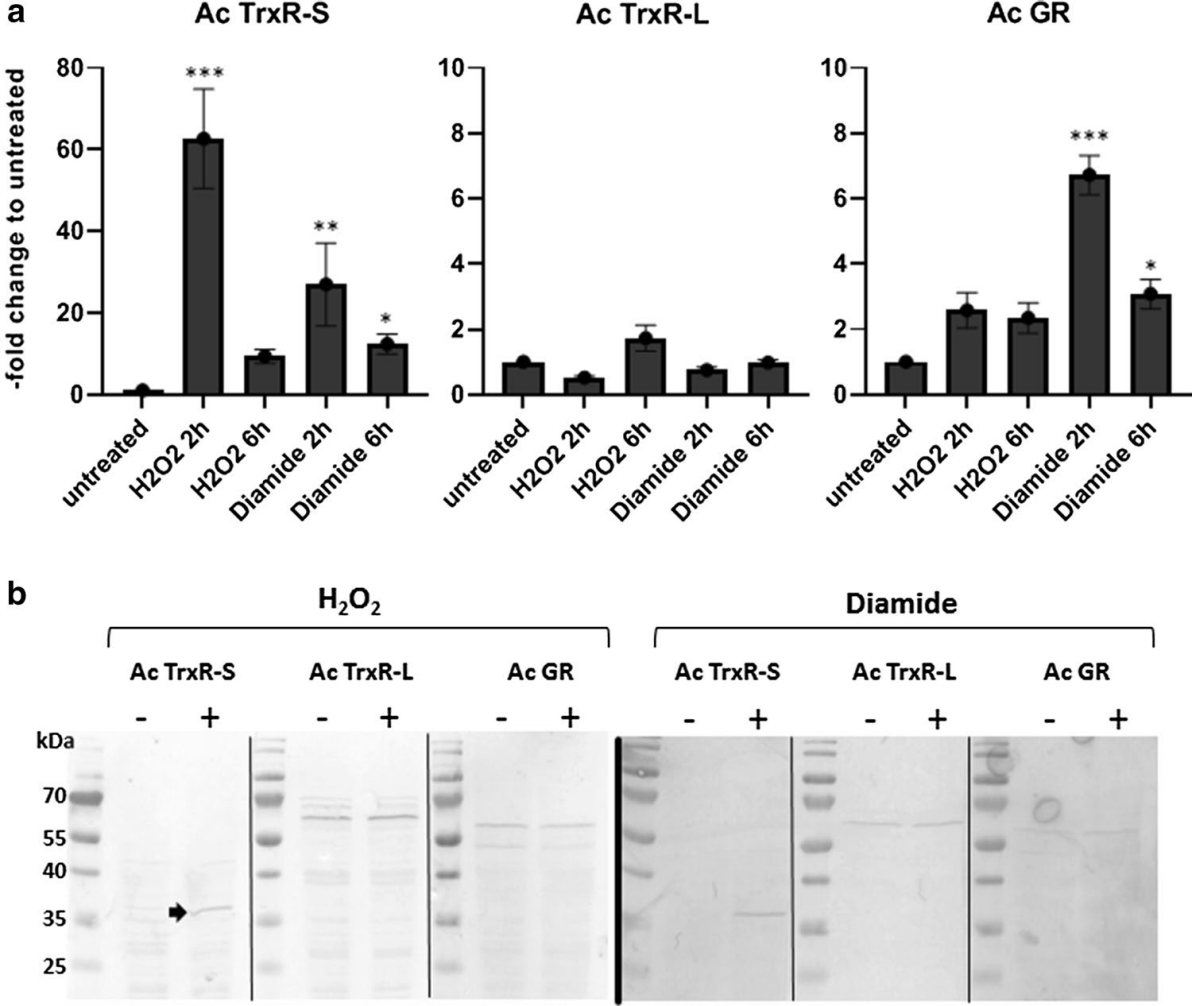
Expression of disulfide reductases in *A*. *castellanii* Neff. **a** The mRNA levels of Ac TrxR-S, Ac TrxR-L and Ac GR were determined by qPCR using 18S RNA and hypoxanthine–guanine phosphoribosyltransferase mRNA as internal standards. Expression levels in untreated controls were compared with expression levels after addition of H_2_O_2_ (750 µM) and diamide (2 mM) for the time periods indicated. The *y*-axis indicates –fold increase of mRNA levels as compared to untreated controls. Error bars show the standard error of the mean (SEM). All values were obtained from three biological replicates in triplicates. * indicates *p* < 0.05, ** indicates *p* < 0.01, and *** indicates *p* < 0.0001 according to the statistical analysis (multiple comparisons with a Kruskal–Wallis test on ranks followed by a Dunn’s post hoc test). **b** Western blots with purified α-Ac TrxR-S (rabbit; dilution 1:100), α-Ac TrxR-L (mouse; dilution 1:100), and α-AcGR (rabbit, dilution 1:100) and cell extracts (equivalent to 50 µg protein) from untreated control cells, and from cells treated either with 750 µM H_2_O_2_ for 18 h (left panel) or 2 mM diamide for 18 h (right panel). The predicted sizes of the disulfide reductases are 34.3 kDa (Ac TrxR-S), 57.7 kDa (Ac TrxR-L), and 48.9 kDa (Ac GR). The thin black lines separating the lanes in each panel indicate that samples were run together on one gel and blotted together on a PVDF membrane, but that the blots were developed independently

TrxRases and GR are major factors in the antioxidative defense, so it was tested which enzyme would be upregulated upon oxidative stress. The expression levels of all three disulfide reductases were determined at the mRNA and protein levels upon addition of hydrogen peroxide (H_2_O_2_), an oxidant readily encountered by acanthamoebae in the host and in their environmental habitats, and of diamide. Diamide oxidizes thiols and causes disulfide stress which can be resolved by disulfide reductases [25]. The mRNA levels of the three disulfide reductases were measured by qPCR as described previously [26]. For the determination of protein expression levels, polyclonal antibodies were raised against each enzyme. To minimize background staining, antibodies were further purified from sera via preparative western blots using the corresponding recombinant enzymes as baits. Expression levels of Ac TrxR-S in untreated cells were very low but the enzyme was strongly upregulated upon addition of H_2_O_2_ and diamide, both at the mRNA and the protein level (Fig. 5). Ac TrxR-S mRNA was upregulated approximately 50-fold at 2 h after addition of H_2_O_2_ with a marked decrease at 6 h (Fig. 5a). The effect of diamide was similar, if less pronounced. At the protein level H_2_O_2_ and diamide induced a strong increase of Ac TrxR-S but not of Ac TrxR-L in a significant manner. Figure 5b is representative for several experiments giving the same result (Supplementary Fig. 3). Treatment with diamide resulted in a similarly pronounced upregulation of Ac TrxR-S as observed with H_2_O_2_ but, again, not of Ac TrxR-L. These observations on the expression of Ac TrxR-S suggested that Ac TrxR-S activity could be measurable in cell extracts of H_2_O_2_ or diamide-treated cells as opposed to untreated cells. However, this was not the case as no TrxR activity could be measured in cell extracts even after treatment of cells with H_2_O_2_ or diamide (not shown). The reason for this is unknown but we had observed earlier with *E*. *histolytica* (unpublished results) that TrxR activity cannot always be measured in cell extracts although the enzyme is clearly expressed.

The expression of GR was far less affected than that of Ac TrxR-S upon oxidative stress. Hydrogen peroxide, and to a larger extent diamide (Fig. 5a), significantly induced levels of Ac GR mRNA (up to sevenfold at 2 h after addition of diamide), but no effect on protein levels could be observed with either stressor (Fig. 5b and Supplementary Fig. 3).

### Intracellular localization of Ac TrxR-L, Ac TrxR-S, and Ac GR in *A*. *castellanii* Neff

**Fig. 6:**
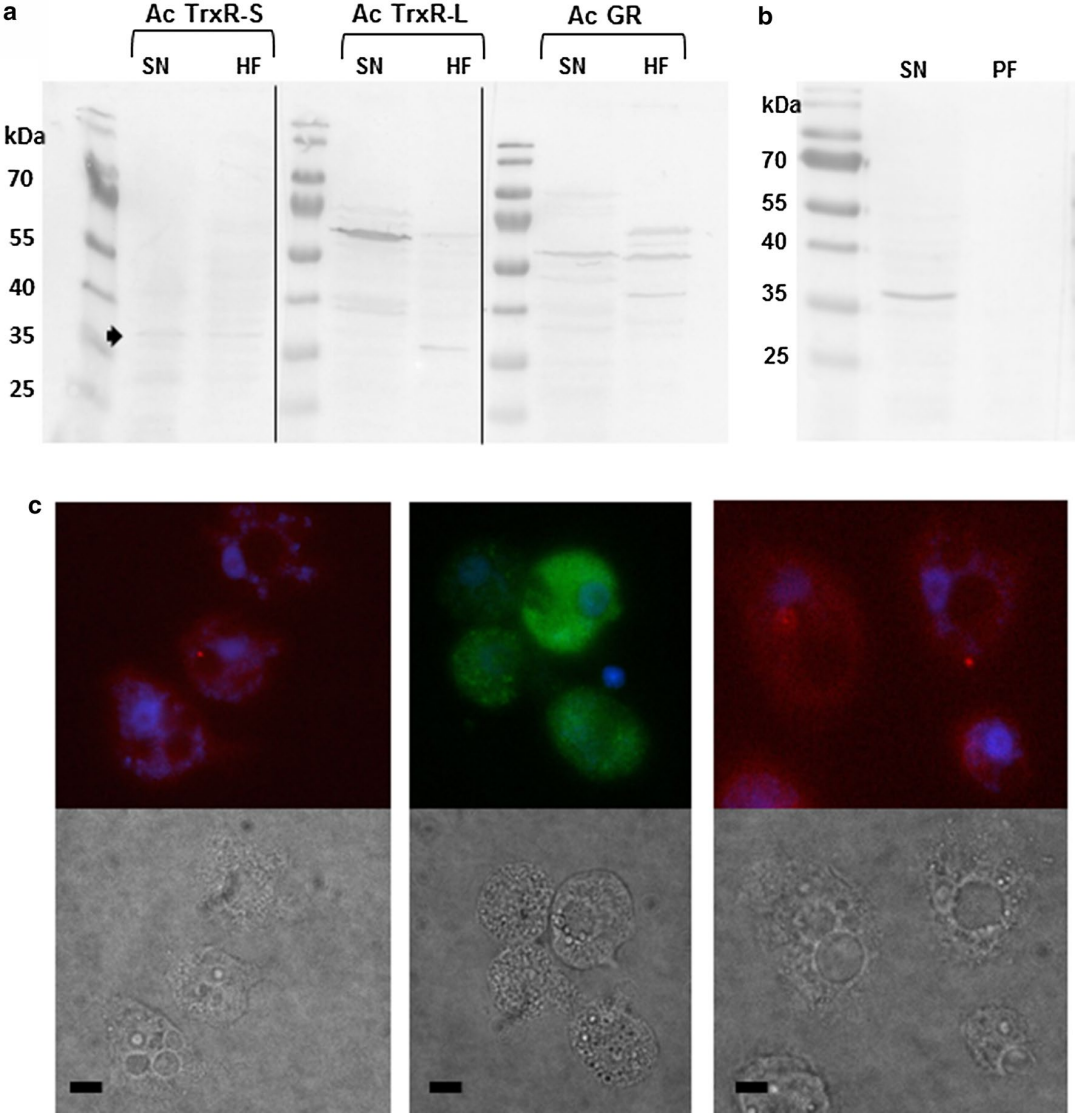
Intracellular localization of the three disulfide reductases in *A*. *castellanii* Neff. **a** Western blots of fractions obtained through subcellular fractionation of *A*. *castellanii* cell extracts with purified antibodies (dilution 1:100) against either disulfide reductase. The loaded amount of protein was 100 µg for both, supernatant (SN) and the heavy fraction from a Percoll gradient (HF) [42]. The additional bands in the pellet fraction obtained with α-Ac GR-antibody might refer to alternatively spliced and partially degraded Ac GR. The arrow indicates the position of Ac TrxR-S. The thin black lines separating the lanes indicate that samples were run together on one gel and blotted together on a PVDF membrane, but that the blots were developed independently. **b** Western blot with α-Ac TrxR-S (dilution 1:100) of supernatant (SN) and pelleted fractions (PF), obtained by centrifugation (4 °C, 20,000 × g, 10 min) of cell lysates of *A*. *castellanii* Neff cells after treatment with 750 µM H_2_O_2_ for 18 h. The latter fraction contains all larger organelles. **c** Immunofluorescence images (1000 × magnification) of *A*. *castellanii* Neff cells after staining with purified antibodies (dilution 1:200) against each of the disulfide reductases. Secondary antibodies (dilutions 1: 10,000) were either conjugated with Alexa Fluor 488 (rabbit anti-Mouse IgG for Ac TrxR-L) or with TRITC (goat anti-rabbit IgG for Ac TrxR-S and Ac GR). Nuclei were stained with DAPI (blue). Light microscopic images of the same cells are given below the according fluorescence images. Scale bar = 5 µm

TrxRases can localize to various intracellular compartments, mainly the cytosol and mitochondria [4]. Eukaryotic GR resides typically in the cytosol and in mitochondria [24]. We wanted to identify the localization of all three disulfide reductases in *A*. *castellanii* and conducted a subcellular fractionation experiment in Percoll gradients. After gentle but complete lysis of cells in a Dounce homogenizer, lysates were centrifuged at 20,000×*g* for 10 min to give a supernatant fraction, consisting predominantly of the cytosol, and a pellet comprising the larger organelles. The fractionation of this pellet by ultracentrifugation in a 37.5% Percoll gradient gave two distinct bands. The lower one, i.e., the heavy fraction, had a brownish color indicating the presence of iron-containing mitochondria. Western blots with the purified antibodies demonstrated (Fig. 6a) that Ac TrxR-L and Ac TrxR-S both reside in the supernatant fraction. In case of Ac TrxR-S there was also a very faint band visible in the heavy fraction of the pellet. However, the overall expression level of this enzyme is very low in the absence of oxidants (Fig. 5b), casting doubt on the relevance of this observation. Therefore, levels of TrxR-S were also compared between the supernatant and pelleted fractions after previous after exposure of cells to H_2_O_2_ (Fig. 6b). We argued that TrxR-S would be strongly upregulated after exposure to H_2_O_2_ (Fig. 5), facilitating the identification of its cellular localization. Indeed, the result clearly showed that Ac TrxR-S is associated with the supernatant fraction and therefore a cytosolic enzyme. Ac GR was found to localize to the cytosol and the heavy fraction of the Percoll gradient alike (Fig. 6a). The upper band from the Percoll gradient, consisting of the light fraction, contained neither disulfide reductase (data not shown).

Since no established cytological markers in *A*. *castellanii* are available to monitor breakage of organelles during the fractionation procedure, we used the same purified antibodies for immunofluorescence microscopy to confer further credibility to above-stated findings. Indeed, the immunofluorescence images confirmed these as all three disulfide reductases were found to be distributed throughout the cell, arguing for a cytosolic localization (Fig. 6c). It can be, therefore, concluded that Ac TrxR-L and Ac TrxR-S are cytosolic enzymes. Ac GR resides in the cytosol and in the organelle fraction (Fig. 6a), as is regularly observed with GR from other organisms [24].

## Discussion

In this study, we showed that *A*. *castellanii* expresses two different types of TrxR, a small bacterial-type TrxR (Ac TrxR-S) and a large vertebrate-type TrxR (Ac TrxR-L) (Table 1). This combination is extremely rare and is only predicted to exist in one other organism known, the free-living amoeba *Naegleria fowleri* [12] which, however, is not closely related to *A*. *castellanii*. To the best of our knowledge, the present study is the first to address function, localization and expression of these TrxRases when occurring in said combination. Both enzymes are cytosolic, whereas Ac GR localizes to the cytosol and the heavy organelle fraction as isolated in Percoll gradients (Fig. 6). In accordance with its surmised function, Ac TrxR-S was found to be strongly upregulated upon oxidative stress (Fig. 5) after exposure to H_2_O_2_ and diamide. Further, Ac TrxR-S reduces Trx-1 which, in turn, reduces Prx-1 and MsrA suggesting an important role of the Ac TrxR-S in the response to oxidative stress. Since Ac TrxR-S was strongly inhibited by auranofin (Table 3), a next-generation antiparasitic compound [7], it is interesting to speculate on Ac TrxR-S being a valid drug target in *A*. *castellanii*. To date, TrxR is the only well-defined drug target of auranofin in protist parasites [21–23]. In contrast, Ac TrxR-L showed no differential expression pattern (Fig. 5) after exposure to oxidative stress, neither at the mRNA level nor at the protein level. Observations from this study further suggest that the steady-state expression level of Ac TrxR-L must be low because the enzyme does not form a clearly discernible spot in Coomassie-stained 2D-gels. For comparison, TrxRases in other protist parasites such as *E*. *histolytica* [27], *Trichomonas vaginalis* [28], and *G*. *lamblia* [29] are readily visible in 2D-gels stained with Coomassie Brilliant Blue, suggesting that expression levels are several orders of magnitude higher. In light of these data, it seems unlikely that Ac TrxR-L is involved in the antioxidant defense under normal growth conditions. Intriguingly, however, the phylogenetically non-related free-living amoeba *N*. *fowleri* also encodes a vertebrate-type TrxR [12] with a UGA codon and a SECIS element. As is the case with *Acanthamoeba*, *N*. *fowleri* can cause severe brain infections, mostly with a fatal outcome. Thus, the vertebrate-type TrxR might be an important pathogenicity factor in both amoebae and could be involved in the response to oxidative or nitrosative stress as exerted by immune cells [30, 31]. Further studies, however, will be necessary to address this hypothesis.

Although direct proof for the insertion of selenocysteine into the C-terminal catalytic site of Ac TrxR-L is missing due to the absence of the C-terminal peptide from the peptides obtained after tryptic digest of isolated Ac TrxR-L (Fig. 3), there is strong circumstantial evidence that Ac TrxR-L has a selenocysteine in its C-terminal catalytic site. All features necessary for the insertion of selenocysteine into Ac TrxR-L are present in *A*. *castellanii*: a SECIS element in the 3′UTR of the Ac TrxR-L gene (Fig. 1), a selenocysteine tRNA and the selenocysteine-specific elongation factor SelB [32]. Unfortunately, this had negative repercussions on our efforts to express functional Ac TrxR-L because this specialized machinery is absent from standard *E*. *coli* expression strains. Furthermore, no expression of recombinant Ac TrxR-L could be observed in the transfected *A*. *castellanii* Neff cell line harboring an expression plasmid with the Ac TrxR-L gene and an intact 3′UTR containing the SECIS element. In contrast, recombinant Ac TrxR-L was strongly expressed when the SECIS element had been scrambled (Fig. 3). This strongly suggests that the absence of selenocysteine in the C-terminal catalytic site renders the enzyme inactive so that the cell can tolerate high levels without being negatively affected. As a prospect, however, expression of functional Ac TrxR-L might be achieved in the specially engineered *E*. *coli* strain C321.ΔA [33] in the future.

In addition to the Trx system, *A*. *castellanii* also encodes a number of components of the glutathione system, most importantly Ac GR. In addition, two glutaredoxins (ACA1_065240 and ACA1_065740) and a glutathione peroxidase (ACA1_365760) are predicted to exist. The latter is a homolog of Hyr1 but does not contain a UGA codon which would be indicative of a selenoprotein. As the focus of this study lay on the Trx system, GR was the only factor to be expressed and assayed for function and localization in the cell. Since Ac GR proved to be highly deleterious when expressed in *E*. *coli*, the enzyme was expressed in *G*. *lamblia*, an anaerobic protist lacking glutathione. GR activity was readily measurable in cell extracts of *A*. *castellanii* Neff as well as *G*. *lamblia* expressing recombinant Ac GR (Fig. 2). The kinetic characteristics of the recombinant enzyme (Table 3) and the dual cellular localization of Ac GR in the cytosol and the heavy organelle fraction (Fig. 6) were comparable to GRases studied before [24]. As observed in other organisms such as *Saccharomyces cerevisiae* [3], the functions of the Trx and glutathione systems might partially overlap. This might explain why the expression levels of TrxRases in *A*. *castellanii* are much lower than in the aforementioned protist parasites which all lack a glutathione system. Quite similar to Ac TrxR-L, the expression levels of Ac GR did not change to a relevant degree upon oxidative stress (Fig. 5), but the high activity of GR as measured in cell extracts indicates that steady-state expression levels of the enzyme must be high.

To summarize, this study is an account on the first in-depth research enterprise focusing on the unusual redox system of the facultative parasite *A*. *castellanii*. Future research may address the function and role of Ac TrxR-L in physiology and pathogenicity and the exploitability of both TrxRases as drug targets. The latter aspect is pressing because anti-acanthamoebal therapy is still difficult and prone to failure.

## Materials and methods

### Chemicals

5,5′-Dithiobis(2-nitrobenzoic acid) (DTNB) was purchased from Merck. NADPH, DL-methionine sulfoxide and oxidized glutathione (GSSG) were purchased from Sigma whereas diamide was purchased from Santa Cruz Biotechnology. Growth media components were either bought from Merck (yeast extract, peptone from casein, sodium chloride), from Sigma (proteose peptone, fetal bovine serum). The TrxR inhibitors auranofin and aurothioglucose were purchased from Sigma.

### Cell culture

*Acanthamoeba castellanii* Neff (ATCC 30,010) cells were grown at RT in PYG (proteose peptone, yeast extract, glucose) medium in Nunc™ EasYFlask™ 75 cm^2^ flasks (Thermo Scientific) and subcultured twice a week (dilution 1:5). *Giardia lamblia* WB C6 (ATCC 50,803) cells were grown in Keister`s modified TYI-S-33 medium containing bile [34] at 37 °C in completely filled Nunclon Delta tubes (Thermo Scientific) and subcultured every second day.

### Recombinant expression of antioxidative enzymes and thioredoxins in *E*. *coli*

Prior to the synthesis of cDNA, RNA was isolated from *A*. *castellanii* Neff cells (GeneJET RNA purification kit, Thermo Fisher) followed by a DNAse I (Thermo Fisher) digest to remove genomic DNA. Reverse transcription was done with the Maxima First strand cDNA kit (Thermo Fisher) according to the manufacturer’s protocol. Genes of interest were subsequently amplified from cDNA by PCR (HOT FIREPol, Solis Biodyne) using primers encoding appropriate restriction sites for insertion into the multiple cloning site of the pET-17b expression vector and a C-terminal 6 × His tag. A summary of the primers used is given in Supplementary Table 3. After cloning of the genes into the expression vector, expression was performed in an appropriate *E*. *coli* expression strain. Both TrxRs, Trx-1, Trx-2, MsrA and MsrB were expressed in BL21-AI™ cells (Thermo Fisher) which express T7 RNA polymerase after induction with L-arabinose. Expression was performed at 37 °C with vivid shaking for 3 h. Peroxiredoxin 1 and 2 (Prx-1, -2) could not be expressed in BL21 AI™ in appreciable amounts due to solubility issues. However, Prx-1 could be expressed over-night in *E*. *coli* Origami™ B(DE3)™ (Novagen) at 18 °C under vivid shaking after induction with 1 mM IPTG, whereas Prx-2 could be expressed ON at 18 °C under vivid shaking in *E*. *coli* SHuffle® T7 cells (New England Biolabs). After expression, *E*. *coli* cultures were spun down and lysed by grinding in a cold (-20° C) mortar with a pestle. After removal of cell debris (20,000 × g, 10 min), recombinant proteins were isolated in Ni-NTA spin columns (Qiagen) via the 6 × His tag.

### Recombinant expression of A. *castellanii* glutathione disulfide reductase (Ac GR) in *G*. *lamblia*

The *A*. *castellanii* GR gene (Ac GR) was amplified from *A*. *castellanii* Neff cDNA and cloned into the pPac-VInteg shuttle vector [35] via PacI and XbaI restriction sites included in the primer sequences. Prior to the gene, the forward primer also contained 50 bp of the 5′UTR of the *G*. *lamblia* arginine deiminase gene (ADI) which constitutes a very strong promoter. The reverse primer encoded a 6 × His tag. Primer sequences are given in Supplementary Table 3. The resulting expression plasmid, pADI-AcGR, was transfected into *G*. *lamblia* WB C6 cells by electroporation in a BTX Electro cell manipulator 600 (Harvard Apparatus) with settings (500 V, 800 µF, and 720 Ω) as used previously in another study [36]. Transfected cells were selected with puromycin (100 µg ml^−1^).

For large-scale expression and isolation of Ac GR, a large NuncTM TripleFlaskTM (Thermo Fisher) was completely filled with *Giardia* growth medium and seeded with approximately 50 × 10^6^ transfected cells (750 ml in total). After growth over-night at 37 °C, cells were harvested (900 × g, 10 min) and lysed by grinding in a cold mortar (− 20 °C) with a pestle. Cell debris was removed (20,000×*g*, 10 min) and the supernatant loaded on a NiNTA superflow column 1.5 ml (Qiagen) for the isolation of 6 × His-tagged AcGR according to the manufacturer’s protocol. Elution of the protein was done in 100 mM sodium phosphate buffer pH 7.4 with 500 mM imidazole. Eluate fractions were tested for GR activity (see below) and the three most active fractions were combined and used for ensuing enzyme assays.

### Expression of Ac TrxR-L in *A*. *castellanii* Neff

A truncated TrxR-L gene terminating at UGA as a stop codon, thus giving rise to a polypeptide lacking the final selenocysteine and glycin residues, was cloned into the pGAPDH vector developed for *A*. *castellanii* [17]. To that end, the enhanced green fluorescent protein (EFGP) gene was excised via the restriction sites NdeI and XhoI and replaced by the truncated TrxR-L gene which had been amplified by PCR using primers bearing the same restriction sites (Supplementary Table 3). The resulting plasmid was transfected into *A*. *castellanii* Neff according to a protocol developed for transfection of *E*. *histolytica* [37]. Briefly, cells were harvested and washed twice in ice-cold PBS and once in electroporation buffer (120 mM KCl, 0.15 mM of CaCl_2_, 25 mM Hepes, 2 mM EGTA, 5 mM MgCl_2_ and 10 mM K_2_HPO_4_). Subsequently, cells were resuspended in 600 µl of electroporation buffer supplemented with 20 µg of the DEAE-dextran. Half of the suspension volume was transferred into a 0.4 cm electroporation cuvette (VWR) containing 100 µg of plasmid. The other half was transferred into another cuvette, without plasmid, and used as a negative control. Electroporation was performed under conditions of 300 V, 800 µF, and 125 Ω. Cells were incubated immediately on ice for 5 min and submitted to a second electroporation pulse under the same conditions. Afterwards, cells were allowed to recover in PYG-medium for 24 h at RT, followed by the selection of transfectants with G-418 (100 µg ml^−1^). After appearance of G418-resistant cells, the medium was replaced. Transfected cells were grown without any antibiotics for 1 week and then subjected to another selection in the same medium containing 50 µg ml^−1^ of G-418.

Full-length Ac TrxR-L including flanking NdeI and XhoI restriction sites, an N-terminal 6 × His-tag after the initiating Met codon, and 381 bp of the 3′ UTR containing the prospective SECIS element was synthesized at Eurofins Genomics (https://www.eurofinsgenomics.eu). The same procedure was repeated with a construct having a scrambled SECIS in the 3′UTR. The sequences of the gene constructs are given in Supplementary Fig. 4. The gene constructs were cloned into the pGAPDH vector and transfected into *A*. *castellanii* Neff as described above. After selection, transfectants were grown in the presence of 1 µM sodium hydrogen selenite (abcr) to provide cells with additional selenium. 150 ml of densely grown culture (app. 2 × 10^8^ cells) were harvested (900 × g, 10 min) and recombinant Ac TrxR-L was isolated via its N-terminal 6 × His-tag as described previously for Ac GR in *G*. *lamblia*.

### Biochemical assays

All measurements involving Ac TrxR-S and Ac TrxR-L were done in 100 mM potassium phosphate buffer pH 6.8 at RT (effectively 21–23° C in an air-conditioned laboratory). TrxR activity was measured along the lines of a published protocol [28] by monitoring thioredoxin (Trx)-dependent reduction of DTNB (1 mM) at *λ* = 412 (Δ*ε*_412_ = 13.6 mM^−1^ cm^−^1) in a spectrophotometer (Lambda 25 UV/Vis, Perkin Elmer) after addition of TrxR (1 µg ml^−1^, equaling 31 nM), Trx-1 (1, 3, 5, 7.5, 10, and 15 µg ml^−1^, equaling 95, 285, 475, 715, 950, and 1185 nM) or Trx-2 (5, 10, 20, 40, and 60 µg ml^−1^, equaling 170, 340, 680, 1360, and 2040 nM), and NADPH. All concentrations of Trx were measured three times. Components were always added in the following order: TrxR – Trx – DTNB – NADPH. Background reduction of DTNB was measured three times by omitting Trx from the mixture. Background values were subtracted from all values obtained with Trx before calculating kinetic parameters. For the determination of the Km of Trx 0.2 mM NADPH were applied, and for the determination of the Km of NADPH (measured at concentrations 2, 3, 6.25, 12.5, and 25 µM), 10 µg ml^−1^ Trx-1 was applied. All concentrations of NADPH were measured three times. For enzyme inhibition studies, inhibitors were added to the reaction mixture [5 µg ml^−1^ Ac TrxR-S (155 nM), 20 µg ml^−1^Trx-1 (1.9 µM), 0.2 mM NADPH, 1 mM DTNB] in varying concentrations (auranofin: 0, 62.5,125, 250, 500, and 1000 nM; aurothioglucose: 0, 0.5, 1, 2, 3, and 5 µM). Auranofin was measured three times at all concentrations given, aurothioglucose only twice for financial reasons.

Trx-dependent peroxidase (Prx) activity was measured as described before [28] at λ = 340 (Δε_340_ = 6.2 mM^−1^ cm^−1^) by monitoring oxidation of NADPH (0.2 mM) by TrxR upon addition of H_2_O_2_ (added at concentrations of 10, 25, 50, 100, 200, and 300 µM). All concentrations of H_2_O_2_ were measured three times and background activity measured without H_2_O_2_ (also three times) was subtracted from all values. The reaction buffer contained 5 µg ml^−1^ of TrxR (155 nM), 20 µg ml^−1^ of Trx (1.9 µM), and 20 µg ml^−1^ of Prx 1 (1 µM) or 2 (1 µM). Methionine sulfoxide reductase (Msr) activity was measured based on a published protocol [38] at *λ* = 340 by monitoring oxidation of NADPH (0.2 mM) by TrxR (5 µg, 155 nM) after addition of DL-methionine sulfoxide (1, 3, 5, 10, and 20 mM) (purchased from Sigma-Aldrich) in the presence of 20 µg ml^−1^ Trx-1 (1.9 µM) and 10 µg ml^−1^ of MsrA or B (610 nM and 715 nM, respectively). All measurements were done three times and the background value measured without DL-methionine sulfoxide (also measured three times) was subtracted from all values. Kinetic parameters and inhibition constants were calculated using GraFit 7 software (Erithacus Software).

Glutathione reductase activity was measured as glutathione (GSH)-dependent reduction of DTNB, analogously to TrxR as described above. The reaction buffer (100 mM Tris, pH 7.5) contained 0.5 µg ml^−1^ Ac GR (11 nM). For the determination of the Km of GSSG, 0.2 mM NADPH was applied; whereas for the determination of the Km of NADPH, 1 mM GSSG was applied.

Measurements with cell extracts were performed in the appropriate buffers with 50 µg protein ml^−1^. Cell extracts were prepared by lysing cells in a Dounce homogenizer followed by centrifugation at 20.000 × g at 4 °C for 10 min to remove cell debris and large organelles. Assay buffers contained 0.2 mM NADPH, 1 mM DTNB, and 20 µg of a candidate Trx when measuring TrxR activity or 1 mM GSSG when measuring GR activity, respectively.

### Expression analysis by RT-qPCR

RNA was isolated using the GeneJET RNA Purification Kit (Thermo Fisher Scientific). After DNase I (Roche) treatment, RNA concentration and purity were determined using a NanoDrop spectrophotometer ND1000 (NanoDrop Technologies). Only samples with a 260/280 ratio between 1.8 and 2.0 were used for subsequent analyses. RNA integrity was assessed by 1% agarose gel electrophoresis. For cDNA synthesis, the amount of total RNA was standardized to 1 µg per reaction. First-strand cDNA was synthesized using the Maxima First Strand cDNA Synthesis Kit for RT-qPCR (Thermo Fisher Scientific). cDNA samples were diluted to 10 ng µl^−1^ using diethyl pyrocarbonate (DEPC)-treated water and stored at -80 °C for further processing. RT-qPCRs were performed in a CFX96 thermocycler (Bio-Rad) using Takyon No Rox SYBR Master Mix dTTP Blue (Eurogentec) in 96-well white PCR plates sealed with Absolute qPCR seals (BioRad). Reaction mixtures contained 200 nM of each primer and 70 ng cDNA (7 µl of 10 ng µl^−1^). No-template reactions were run as negative controls. The RT-qPCR temperature profile included an initial denaturation step at 95 °C for 3 min, followed by 45 cycles of 15 s at 95 °C, 15 s at 55 °C and 15 s at 72 °C. Melting curves were determined after RT-qPCR run (65–95 °C). All experiments were carried out in at least three independent set ups. For each cDNA batch, three independent RT-qPCR runs were performed (resulting in total in three biological replicates in triplicate). The relative expression was normalized towards 18S rRNA and hypoxanthine–guanine phosphoribosyltransferase (HPRT) mRNA as reference genes [26]. Primer sequences are shown in Supplementary Table 3.

The number of repetitions was found to be sufficient to demonstrate upregulation of Ac TrxR-S and GR upon oxidative stress in a statistical significant manner. Given the absence of a normal distribution of values due to the small sample size, the statistical significance of the results obtained per gene was tested in multiple comparisons with a Kruskal–Wallis test on ranks followed by a Dunn’s post hoc test (GraphPad 9 software). Adjusted p-values were determined separately for both normalizations (18S rRNA and HPRT, respectively), and only those values that were statistically significant using both normalizations were considered as truly significant. This conservative approach was chosen in order to minimize the occurrence of type I errors.

### Two-dimensional gel electrophoresis (2DE) and mass spectrometry

Densely grown *A*. *castellanii* NEFF cultures (approximately 5 × 10^7^ cells) in late exponential phase were harvested (4 °C, 800 × g for 5 min). Cell pellets were washed once in PBS and once in ultrapure water, respectively, and finally resuspended in 2 ml of 10% trichloroacetic acid in acetone. Protein precipitation was performed at – 20 °C for 1 h. Precipitates were pelleted at 20,000 × g in a cryocentrifuge (4 °C) and washed twice in 90% acetone in ultrapure water. Finally, proteins were resolubilized in 2DE sample buffer containing 7 M urea, 2 M thiourea, 4% 3-[(3-cholamidopropyl)dimethylammonio]-1-propanesulfonate hydrate (CHAPS, Sigma), 1% ampholytes Biolyte pH 3–10, BioRad) and 1% dithiothreitol (DTT, Sigma). Insoluble material was pelleted (20,000 × g at 20 °C for 20 min) and supernatants were used for 2DE. Protein concentrations in samples were determined by Bradford assay and 500 mg of protein was loaded for isoelectric focusing in 17 cm IPG strips (BioRad). Isoelectric focussing and second-dimension PAGE were performed as described before [39]. 2D-gels were stained with Coomassie Brilliant-Blue R250 and relevant protein spots were excised.

The submitted protein spots were treated according to standard protocols [40]. Subsequently, in-gel digestion of the proteins was performed in 50 mM aqueous ammonium bicarbonate and 5 mM CaCl_2_ with trypsin (Trypsin Gold, Mass Spectrometry Grade, Promega, Madison, WI) at a concentration of 20 ng µl^−1^ for 8 h at 37 °C [41]. Afterwards, peptides were extracted and dried down. After resuspension of the peptides in 0.1% TFA, LC–MS/MS analysis was performed. Peptides were separated on a nano-HPLC Ultimate 3000 RSLC system (Dionex). Separation of peptides was performed in a 25 cm Acclaim PepMap C18 with direct coupling to a high-resolution Q Exactive HF Orbitrap mass spectrometer and mass spectrometric full scans were performed in the ultrahigh-field Orbitrap mass analyzer. Peptide masses were matched against NCBI and Swiss Prot databases.

### Antibodies and western blotting

Antibodies against AcTrxR-S (rabbit), AcTrxR-L (mouse) and AcGR (rabbit) were produced by GenicBio Limited (Shanghai) against the peptides LWVEGEEEGEPAA (AcTrxR-S), KYPDIPGDREFGITS (AcTrxR-L), and KLIDNKDKEIDRLN (AcGR). Antibodies were further purified from antisera along the lines as described [36] using the respective recombinant proteins as baits on a PVDF membrane and stripping off the bound antibodies after incubation. These additionally purified antisera were highly specific for the respective protein and could be used for quantitative measurements of expression levels. SDS-PAGE and western blotting (onto PVDF membranes) were performed according to standard protocols in a Mini Protean® Tetra Cell (BioRad). Blots were developed using alkaline phosphatase-linked secondary antibodies from Sigma (goat anti-Rabbit IgG, A3687; and goat anti-mouse IgG, A3562, respectively).

### Subcellular fractionation of *A*. *castellanii* lysates

After harvest, acanthamoebae were lysed gently in a Dounce homogenizer in the presence of protease inhibitors (protease inhibitor cocktail for use with fungal and yeast extracts, Sigma). Prior to subcellular fractionation, non-lysed amoebae were pelleted and removed by centrifugation at 300×*g* for 3 min. Subcellular fractionation of *A*. *castellanii* cell lysates was performed in Percoll gradients as described previously [42].

### Immunofluorescence microscopy

*A*. *castellanii* Neff cells from grown cultures were harvested at 4 °C (1200 × g, 5 min). Pellets were resuspended in 400 µl of PYG (proteose peptone, yeast extract, glucose) medium and applied onto µ-dishes (Ibidi). After attachment, 1.6 ml of medium was added and dishes were covered with supplied lids and left for incubation overnight at RT. Afterwards, cells were washed twice with ice-cold PBS and fixed at a concentration of 2% of paraformaldehyde in PBS. After fixation of cells (30 min at 37 °C), the fixative was removed by pipetting, followed by incubation in 0.1 M glycine in PBS (10 min at RT). Subsequently, the cells were permeabilized in PBS + 0.1% TritonX-100 (30 min at RT) and incubated in blocking solution (2% BSA in PBS + 0.05% TritonX-100) for 1 h. Purified primary antibodies (α-TrxR-S, α-TrxR-L and α-GR) were added at dilutions of 1:200 for 1 h. Afterwards, fixed cells were washed six times in PBS and appropriate secondary antibodies were added at dilutions of 1: 10,000 [rabbit anti-Mouse IgG (Thermo Fisher, USA) conjugated with Alexa Fluor 488 targeting TrxR-L and goat anti-rabbit (Novex by life technologies, USA) conjugated with TRITC for TrxR-S and GR]. After incubation for 1 h, dishes were washed six times in PBS and DAPI was added (5-min incubation in darkness) followed by another washing step in PBS. Mounting medium was placed onto dishes and immunofluorescence microscopy was performed in an IX71 microscope at a total magnification of 1000 × .

## Supplementary Information

Below is the link to the electronic supplementary material.

Supplementary file1 (PPTX 340 KB)

Supplementary file2 (PDF 366 KB)

Supplementary file3 (PDF 373 KB)

Supplementary file4 (PDF 250 KB)

Supplementary file5 (PDF 251 KB)

Supplementary file6 (XLSX 62 KB)

Supplementary file7 (PDF 269 KB)

## Data Availability

The datasets generated during and analyzed during the current study are available as supplementary information to this work.
