# Supplementary material for: An unusual thioredoxin system in the facultative parasite Acanthamoeba castellanii
Source: Cell Mol Life Sci. 2021 Feb 18;78(7):3673–89. doi: 10.1007/s00018-021-03786-x (PMC8038987; doi:10.1007/s00018-021-03786-x)
Supplement: Supplementary file 1 — Supplementary file1 (PPTX 340 KB) [file 18_2021_3786_MOESM1_ESM.pptx]

## Slide 1
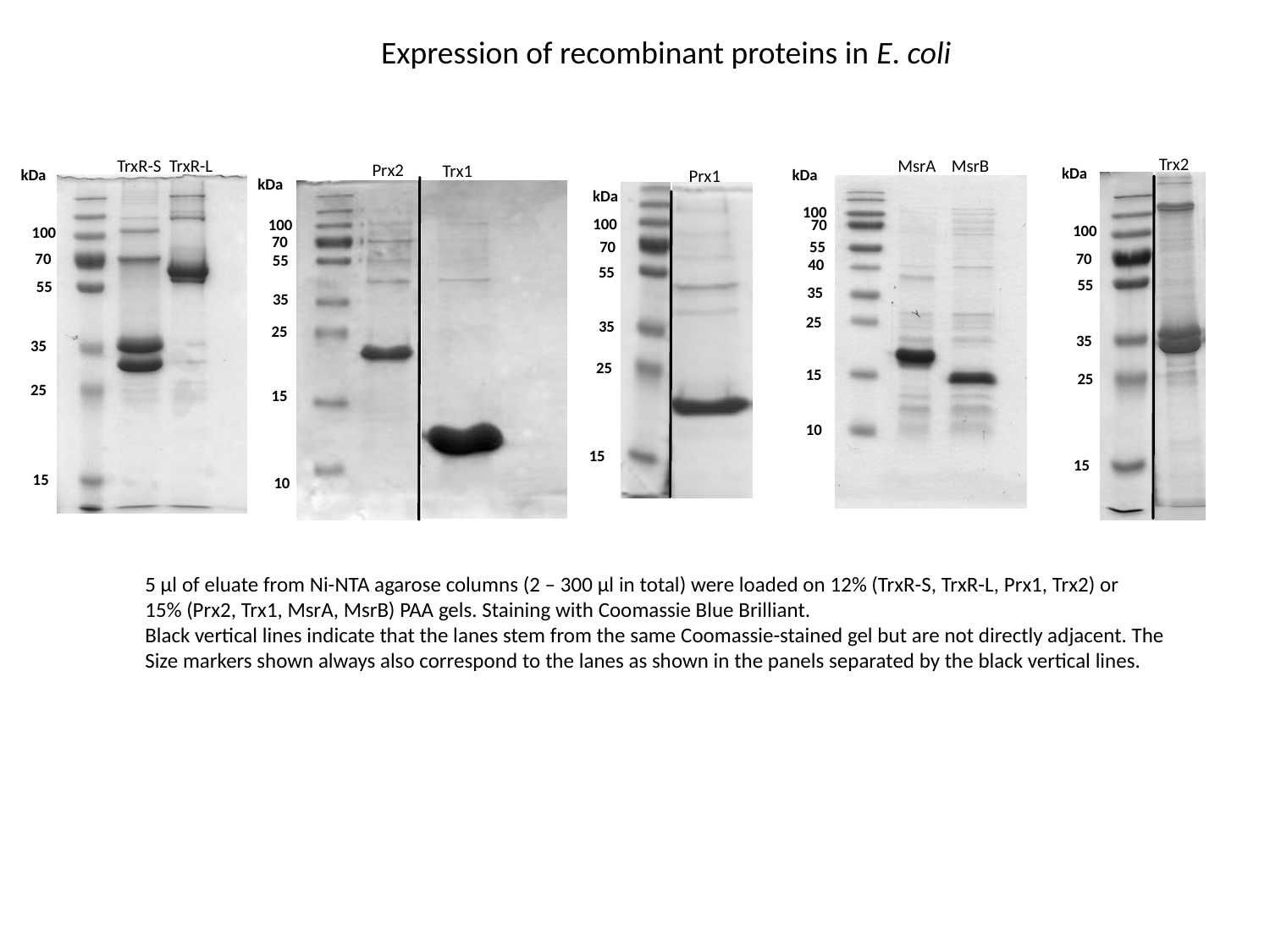

Expression of recombinant proteins in E. coli
Trx2
TrxR-L
TrxR-S
kDa
100
70
55
35
25
15
MsrB
MsrA
Prx2
Trx1
kDa
100
70
55
35
25
15
10
kDa
Prx1
kDa
100
70
55
35
25
15
kDa
100
70
100
55
70
40
55
35
25
35
15
25
10
15
5 µl of eluate from Ni-NTA agarose columns (2 – 300 µl in total) were loaded on 12% (TrxR-S, TrxR-L, Prx1, Trx2) or
15% (Prx2, Trx1, MsrA, MsrB) PAA gels. Staining with Coomassie Blue Brilliant.
Black vertical lines indicate that the lanes stem from the same Coomassie-stained gel but are not directly adjacent. The
Size markers shown always also correspond to the lanes as shown in the panels separated by the black vertical lines.
