# Supplementary figures and images for: An unusual thioredoxin system in the facultative parasite Acanthamoeba castellanii
Source: Cell Mol Life Sci. 2021 Feb 18;78(7):3673–89. doi: 10.1007/s00018-021-03786-x (PMC8038987; doi:10.1007/s00018-021-03786-x)

Supplementary Figure 2: Peptides identified in Ac TrxR-L by mass spectrometry:

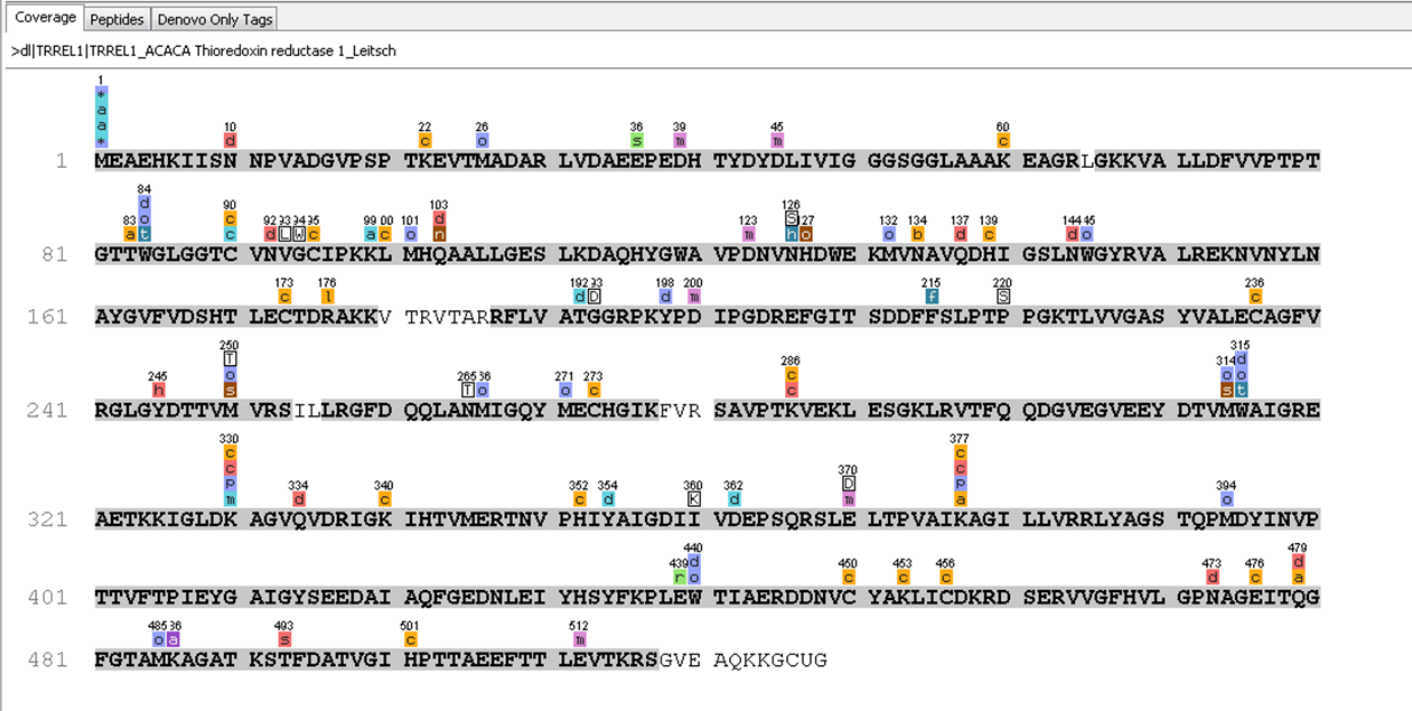

Supplement: Supplementary file 2 — Supplementary file2 (PDF 366 KB) [file 18_2021_3786_MOESM2_ESM.pdf]
