# Supplementary material for: An unusual thioredoxin system in the facultative parasite Acanthamoeba castellanii
Source: Cell Mol Life Sci. 2021 Feb 18;78(7):3673–89. doi: 10.1007/s00018-021-03786-x (PMC8038987; doi:10.1007/s00018-021-03786-x)
Supplement: Supplementary file 3 — Supplementary file3 (PDF 373 KB) [file 18_2021_3786_MOESM3_ESM.pdf]

Supplementary Figure

Ac TrxR-S

Western blots of *A. castellanii* cell extracts with purified α-TrxR-S ab (1:100)
Secondary ab (anti rabbit): 1: 5000.

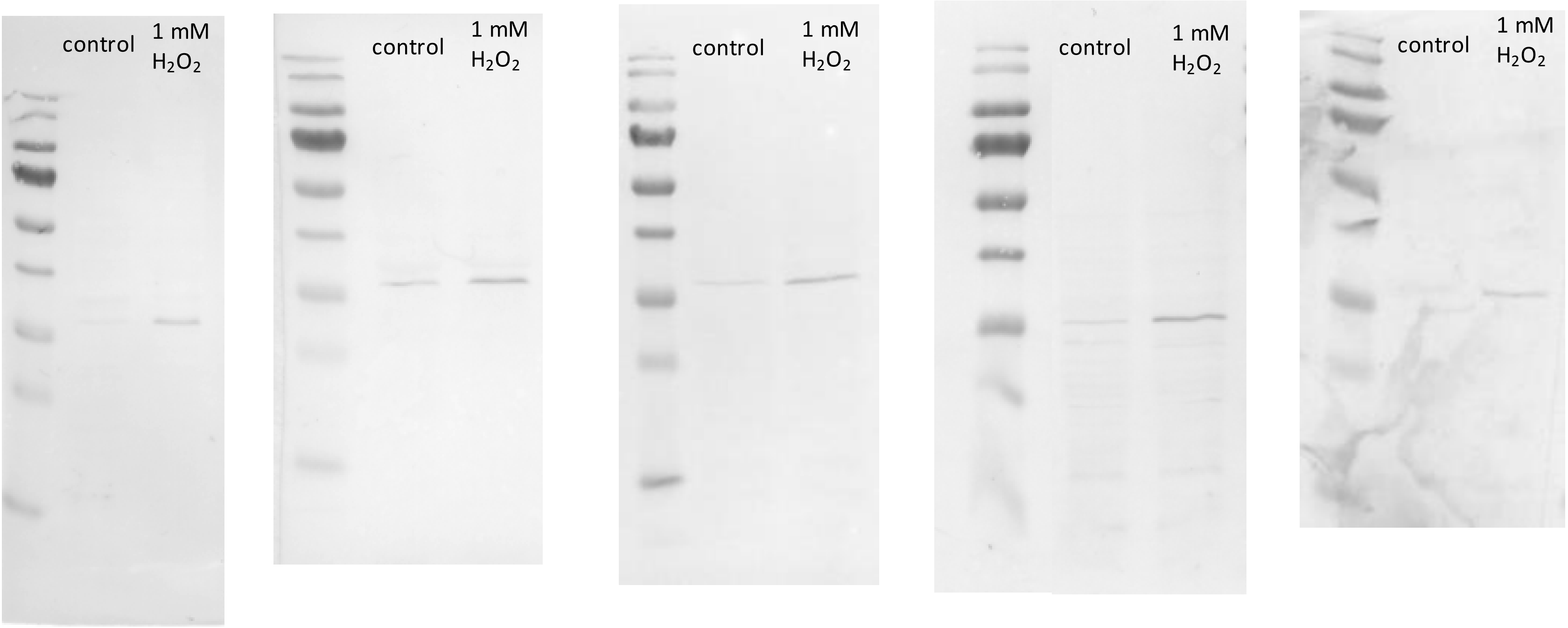

In all experiments 50 µg ml^{-1} protein of *A. castellanii* Neff cell extract were used.

Upregulation of Ac TrxR-S upon H_2O_2 exposure (18 h) can be seen in all instances

Ac TrxR-L

Western blots of *A. castellanii* cell extracts with α-TrxR-L ab (1:100)
Secondary ab (anti mouse): 1: 5000.

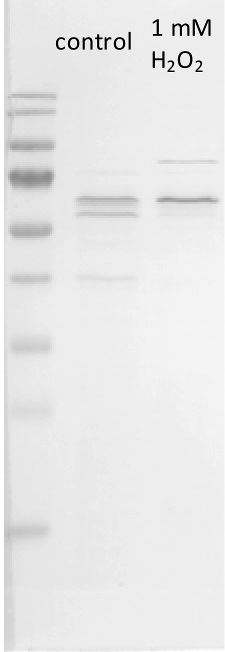

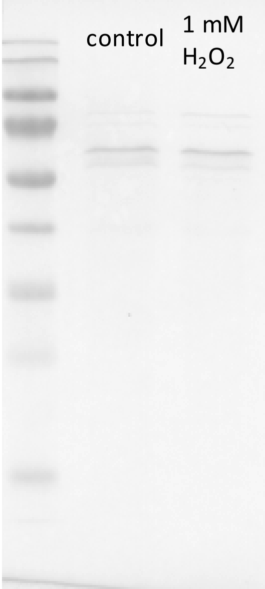

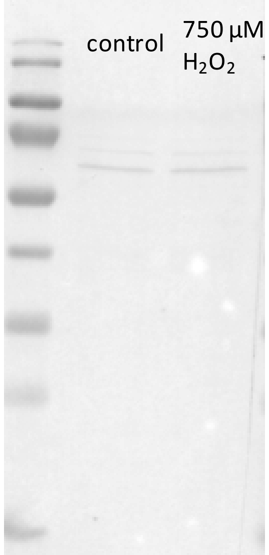

In all experiments 50 µg ml^{-1} protein of *A. castellanii* Neff cell extract were used.

No upregulation of Ac TrxR-L upon H_2O_2 exposure (18 h) can be seen.

Ac GR

Western blots of *A. castellanii* cell extracts with α-GR ab (1:100)
Secondary ab (anti rabbit): 1: 5000.

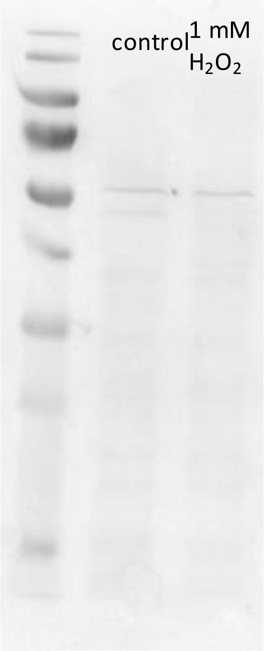

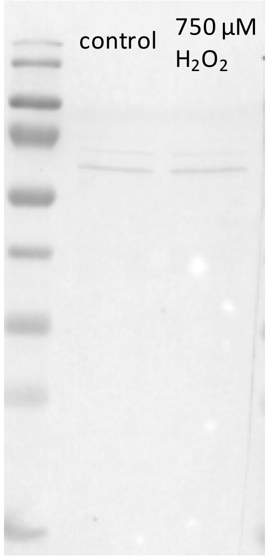

In both experiments 50 µg ml^{-1} protein of *A. castellanii* Neff cell extract were used.

No upregulation of Ac GR upon H_2O_2 exposure (18 h) can be seen.
