# Supplementary material for: An unusual thioredoxin system in the facultative parasite Acanthamoeba castellanii
Source: Cell Mol Life Sci. 2021 Feb 18;78(7):3673–89. doi: 10.1007/s00018-021-03786-x (PMC8038987; doi:10.1007/s00018-021-03786-x)
Supplement: Supplementary file 4 — Supplementary file4 (PDF 250 KB) [file 18_2021_3786_MOESM4_ESM.pdf]

Supplementary Figure 4: Ac TrxR-L construct with SECIS as expressed in *A. castellanii* Neff

1. **Intact** SECIS with N-terminal His tag (cloned via NdeI and XbaI):

CATATGCATCACCATCACCATCACGAGGCCGAACACAAAATCATCTCCAATAATCCGGTAGCAGACGGCGTGC
CGTCCCCCACCAAGGAGGTCACCATGGCCGACGCTCGCTTAGTGGACGCGGAAGAGCCCGAGGACCACACCT
ACGACTACGATCTCATCGTGATCGGCGGCGGCTCTGGTGGTCTCGCCGCGGCCAAGGAGGCCGGCAGGCTGG
GCAAGAAGGTCGCCCTCCTGGACTTTGTCGTGCCCACGCCCACCGGCACCACCTGGGGCCTTGGCGGCACCTG
CGTCAACGTGGGGTGCATTCCGAAGAAGCTCATGCACCAGGCTGCGCTCCTGGGCGAGTCGCTCAAGGACGC
CCAGCATTACGGCTGGGCCGTGCCGGACAACGTGAACCATGACTGGGAGAAGATGGTGAACGCCGTGCAGG
ACCACATTGGGTCGCTCAACTGGGGCTACCGTGTGGCGCTCCGCGAGAAGAACGTCAACTATCTCAACGCCTA
CGGCGTGTTCGTCGATTCGCATACCCTCGAGTGCACCGACCGTGCCAAGAAAGTGACTCGCGTGACGGCGCGC
CGCTTCCTGGTGGCGACCGGCGGCAGGCCCAAGTACCCGGACATCCCGGGCGACCGCGAGTTCGGCATCACC
TCCGACGACTTCTTCTCGCTCCCGACCCCGCCCGGCAAGACGCTCGTCGTGGGCGCGTCGTACGTGGCACTGG
AGTGCGCCGGTTTCGTGCGCGGGCTGGGCTACGACACCACCGTCATGGTGCGATCGATCCTGCTGCGCGGATT
CGACCAACAGCTCGCCAACATGATCGGCCAATACATGGAGTGCCACGGCATCAAGTTCGTGCGGTCCGCCGTT
CCGACCAAGGTCGAGAAGCTCGAGAGCGGAAAGCTGCGGGTCACCTTCCAGCAGGACGGCGTCGAGGGAGT
GGAGGAGTACGACACGGTGATGTGGGCGATTGGCCGCGAGGCCGAGACCAAGAAGATCGGGCTCGACAAG
GCCGGCGTGCAGGTCGACCGCATCGGCAAGATCCACACCGTCATGGAGCGCACCAACGTTCCCCACATCTACG
CCATCGGCGACATCATCGTCGACGAGCCGAGCCAGCGCAGTCTGGAGCTCACGCCCGTCGCCATCAAGGCCG
GCATCTTGCTCGTGCGACGACTCTACGCCGGGTCCACCCAGCCCATGGACTACATCAACGTGCCCACGACGGT
GTTCACGCCCATCGAGTACGGTGCCATCGGCTACAGCGAGGAGGACGCCATCGCCCAGTTCGGCGAGGACAA
CCTCGAGATCTACCACTCGTACTTCAAGCCGCTCGAGTGGACCATCGCCGAGCGGGACGATAACGTGTGCTAC
GCCAAGCTCATCTGCGACAAGCGCGACTCGGAGCGCGTGGTGGGCTTCCACGTCCTCGGCCCCAACGCCGGC
GAGATCACCCAGGGCTTCGGCACTGCCATGAAGGCGGGTGCTACGAAGAGCACGTTCGACGCCACGGTGGGC
ATCCACCCGACTACAGCGGAGGAGTTCACGACGCTCGAAGTTACCAAACGTTCTGGGGTGGAGGCCCAGAAG
AAGGGCTGC**TGA**GGT**TAA**GCTGCTGCCGCCCTCAGCCTCCTCGCTACACACTCCGTCGCGCGCCTTGAGACGC
TCTCAGGCCAGGCCAACGGTGATGCTCAGTCACCAGCGACGGAGAAGAGGAGGCGGGGCTGCAGCAGGTTA
ATCCCTGACGTACTCCACCTCTCCCCCTTCCCCCACATCATCTCTCCGGTCCTCTCCTCCACATCTCTCTTCTTCTC
CTCTCTTTCTCTATCTACGCTCATCTTCCCTCCCACTCACTACGAGGCCGTGAGAGGAGAAGCCTCTTATTCTCTC
CCTCGGCTGTTTGCAAATGACGGCGCCAGCGTAAAGCTCACCACGAGCCCGCTTGCGTCTGACTGTCGAACAC
CACAGCAATAAAGGCTTCTCTTACGATAACAAACTCTAGA

2. **Scrambled** SECIS with N-terminal His tag (cloned via NdeI and XbaI):

CATATGCATCACCATCACCATCACGAGGCCGAACACAAAATCATCTCCAATAATCCGGTAGCAGACGGCGTGC
CGTCCCCCACCAAGGAGGTCACCATGGCCGACGCTCGCTTAGTGGACGCGGAAGAGCCCGAGGACCACACCT
ACGACTACGATCTCATCGTGATCGGCGGCGGCTCTGGTGGTCTCGCCGCGGCCAAGGAGGCCGGCAGGCTGG
GCAAGAAGGTCGCCCTCCTGGACTTTGTCGTGCCCACGCCCACCGGCACCACCTGGGGCCTTGGCGGCACCTG
CGTCAACGTGGGGTGCATTCCGAAGAAGCTCATGCACCAGGCTGCGCTCCTGGGCGAGTCGCTCAAGGACGC
CCAGCATTACGGCTGGGCCGTGCCGGACAACGTGAACCATGACTGGGAGAAGATGGTGAACGCCGTGCAGG

ACCACATTGGGTCGCTCAACTGGGGCTACCGTGTGGCGCTCCGCGAGAAGAACGTCAACTATCTCAACGCCTA
CGGCGTGTTCGTCGATTCGCATACCCTCGAGTGCACCGACCGTGCCAAGAAAGTGACTCGCGTGACGGCGCGC
CGCTTCCTGGTGGCGACCGGCGGCAGGCCCAAGTACCCGGACATCCCGGGCGACCGCGAGTTCGGCATCACC
TCCGACGACTTCTTCTCGCTCCCGACCCCGCCCGGCAAGACGCTCGTCGTGGGCGCGTCGTACGTGGCACTGG
AGTGCGCCGGTTTCGTGCGCGGGCTGGGCTACGACACCACCGTCATGGTGCGATCGATCCTGCTGCGCGGATT
CGACCAACAGCTCGCCAACATGATCGGCCAATACATGGAGTGCCACGGCATCAAGTTCGTGCGGTCCGCCGTT
CCGACCAAGGTCGAGAAGCTCGAGAGCGGAAAGCTGCGGGTCACCTTCCAGCAGGACGGCGTCGAGGGAGT
GGAGGAGTACGACACGGTGATGTGGGCGATTGGCCGCGAGGCCGAGACCAAGAAGATCGGGCTCGACAAG
GCCGGCGTGCAGGTCGACCGCATCGGCAAGATCCACACCGTCATGGAGCGCACCAACGTTCCCCACATCTACG
CCATCGGCGACATCATCGTCGACGAGCCGAGCCAGCGCAGTCTGGAGCTCACGCCCGTCGCCATCAAGGCCG
GCATCTTGCTCGTGCGACGACTCTACGCCGGGTCCACCCAGCCCATGGACTACATCAACGTGCCCACGACGGT
GTTCACGCCCATCGAGTACGGTGCCATCGGCTACAGCGAGGAGGACGCCATCGCCCAGTTCGGCGAGGACAA
CCTCGAGATCTACCACTCGTACTTCAAGCCGCTCGAGTGGACCATCGCCGAGCGGGACGATAACGTGTGCTAC
GCCAAGCTCATCTGCGACAAGCGCGACTCGGAGCGCGTGGTGGGCTTCCACGTCCTCGGCCCCAACGCCGGC
GAGATCACCCAGGGCTTCGGCACTGCCATGAAGGCGGGTGCTACGAAGAGCACGTTCGACGCCACGGTGGGC
ATCCACCCGACTACAGCGGAGGAGTTCACGACGCTCGAAGTTACCAAACGTTCTGGGGTGGAGGCCCAGAAG
AAGGGCTGC**TGA**GGT**TAA**AAGCTGCTGCCGCCCTCAGCCTCCTCGCTACACACTCCGTCGCGCGCCTTGAGAC
GCTCTCAGGCCAGGCCAACGGTGATGCTCAGTCACCAGCGACGGAGAAGAGGAGGCGGGGCTGCAGCAGGT
TAATCCCTGACGTACTCCACCTCTCCCCCTTCCCCCACATCATCTCTCCGGTCCTCTCCTCCACATCTCTCTTCTTC
TCCTCTCTTTCTCTATCTACGCTCATCTTCCCTCCCACTCTGAGCGTAGATAGAGAAAGAGAGGAGAAGCTCTCC
CTCGGCTGGTGGTGTTCGACAGTCAGACGCAAGCGGGCTCGTGGTGAGCTTTACGCTGGCGCCGTCATTTGCA
AACAGCCGAGGGAGAGTCTAGA
