# Supplementary material for: An unusual thioredoxin system in the facultative parasite Acanthamoeba castellanii
Source: Cell Mol Life Sci. 2021 Feb 18;78(7):3673–89. doi: 10.1007/s00018-021-03786-x (PMC8038987; doi:10.1007/s00018-021-03786-x)
Supplement: Supplementary file 5 — Supplementary file5 (PDF 251 KB) [file 18_2021_3786_MOESM5_ESM.pdf]

Supplementary Table 2. Proteins with thioredoxin-like domain which were not considered in this study.

NCBI data base entry	***Reason for exclusion***
XP_004340322	PDI-family
XP_004341090	No cysteines
XP_004347206	PDI-like
XP_004357006	PDI-like
XP_004353435	PDI A6
XP_004352846	PDI-family
XP_004344023	DsbA family
XP_004341546	PDI-like
XP_004339861	PDI-like
XP_004339289	PDI family
XP_004335451	PDI-like
