# Supplementary material for: An unusual thioredoxin system in the facultative parasite Acanthamoeba castellanii
Source: Cell Mol Life Sci. 2021 Feb 18;78(7):3673–89. doi: 10.1007/s00018-021-03786-x (PMC8038987; doi:10.1007/s00018-021-03786-x)
Supplement: Supplementary file 7 — Supplementary file7 (PDF 269 KB) [file 18_2021_3786_MOESM7_ESM.pdf]

Supplementary Table 1: Primers used for expression constructs and RT-qPCR

Protein	**NCBI entry**	**Primer fw**	**Primer rv**
Recombinant expression in E. coli			
Ac TrxR-S	XP_004351681	tacgtacgcatatggagagcaaagtacacagaa	tcatccagctcgagttagtgatggtgatggtgatggaggtgggccgagag
Ac TrxR-L	XP_004353633	tacgtacgcatatggaggccgaacacaaaatc	tcatccaggaattcttagtgatggtgatggtgatggcagcccttcttctgg
Ac TrxR-L<br>UGA replaced with cysteine codon; N-term. His tag	XP_004353633	tacgtacgcatatgcatcaccatcaccatcacgaggccgaacacaaaatc	tcatccaggaattcttaaccgcagcagcccttcttctgg
Ac GR	XP_004338246	tacgtacgcatatgggaaagtgccaagtctac	tcatccagctcgagttagtgatggtgatggtgatgctccctcttggttcgcat
Trx-1	XP_004335509	tacgtacgcatatggtcaagcaggtcaccagc	tcatccagctcgagttagtgatggtgatggtgatgctggtgcttggtgatgcc
Trx-2	XP_004349558	tacgtacgcatatggatcacggcagagttcag	catccaggaattcttagtgatggtgatggtgatggccatgcgtctcgcccttc
Trx candidate; PITH domain	XP_004341439	tacgtacgcatatgaagcgggagcac	tcatccagctcgagttagtgatggtgatggtgatgtttgtcattggtgaggtc
Trx candidate	XP_004337209	tacgtacgcatatggagcttcctcggattcgg	tcatccagctcgagttagtgatggtgatggtgatggctctgggtcgtcttctt
Prx-1	XP_004344639	tacgtacgcatatggacgctcggtttgcg	atccaggaattcttagtgatggtgatggtgatgctgggattgcaccgc
Prx-2	XP_004333640	tacgtacgcatatgtccacccacgccgttat	tcatccagctcgagttagtgatggtgatggtgatgctggttgaccttctcgaag
Prx	XP_004353768	tacgtacgcatatgaagaggaggcgtgacgaa	tcatccagctcgagttagtgatggtgatggtgatgttggtccatgtccgtgtt
Prx	XP_004348542	tacgtacgcatatggggcttgtagcgctgc	catccaggaatccttagtgatggtgatggtgatgcagctggatatccttgaag
MsrA	XP_004342149	tacgtacgcatatgaccgaaaccgctaccttt	catccaggaattcttagtgatggtgatggtgatgccagcggagtcggtgg
MsrB	XP_004367953	tacgtacgcatatgacatcgttccccgtagag	tcatccagctcgagttagtgatggtgatggtgatggtgcttggccttgtcag
Recombinant expression in G. lamblia			
Ac GR	XP_004338246	catctagaaacgtctacacgtgaggtgtgtaaacttccggagaaaaaaatcctagtacatggaaagtgccaagtctac	cattaattaattagtgatggtgatggtgatgctccctcttggttcgcat
Recombinant expression in A. castellanii			
Ac TrxR-L<br>No 3’ UTR<br>Last codon UGA	XP_004353633	tacgtacgcatatggaggccgaacacaaaatc	tcatccagtctagattagcagcccttcttctgg
Primers for qPCR			
Ac TrxR-S	XP_004351681	ctctcgaaccccaagatc	cacctgaccattcaggaac
Ac TrxR-L	XP_004353633	ggattcgaccaacagctcg	gagcccgatcttcttggtc
Ac GR	XP_004338246	cgacactctctacaacaacc	cttctcgtacacgcttggac
18S rDNA		cccagatcgtttaccgtgaa	taaatattaatgcccccaactatcc
HPRT		ggagcggatcgttctctg	atcttggcgtcgacgtgc
